# Reactive Molecular Dynamics Simulations of Proton Transport in PFSA Membranes: Hydration–Temperature Mechanism Crossover and Cold-Start Implications

**DOI:** 10.3390/membranes16100335

**Published:** 2026-10-09

**Authors:** Haibin Lu, Wanhua Qi, Mingzhao Lai, Hui Ye, Taosif Iqbal, Changchen Xie, Yaoli Xu

**Affiliations:** 1 Flight College, Anyang Institute of Technology, Anyang 455000, China; 2 College of Computer Science and Information Engineering, Anyang Institute of Technology, Anyang 455000, China; 3 Electrical Engineering Department, College of Electrical and Mechanical Engineering, National University of Sciences and Technology, Islamabad 44000, Pakistan; 4 Sihe Coal Mine, Jinneng Holding Equipment Manufacturing Group Co., Ltd., Jincheng 048000, China; 5 Software Engineering College, Zhengzhou University of Light Industry, Zhengzhou 450001, China

**Keywords:** proton exchange membrane fuel cell, PFSA membrane, proton transport, Grotthuss mechanism, vehicle mechanism, reactive molecular dynamics, cold start, activation energy

## Abstract

Proton transport through perfluorosulfonic acid (PFSA) membranes sets the ohmic resistance of proton exchange membrane fuel cells and is most constrained during cold start, when reduced hydration coincides with sub-ambient temperatures, yet the conditions under which the Grotthuss and vehicle mechanisms dominate remain unresolved. Here, ReaxFF reactive molecular dynamics with explicit proton-transfer event statistics characterizes this crossover across 48 trajectories spanning λ = 6 and λ = 14 at 230–300 K. At λ = 6, every trajectory shows zero Grotthuss hops at all temperatures and no hydronium-like motif; the acidic proton stays bound to its parent sulfonate, so the nonzero tracer diffusivity reflects localized motion rather than vehicle transport, and the apparent activation energy of 0.09 eV serves as an order-of-magnitude anchor for continuum cold-start models. Because λ = 6 remains hop-free even at 300 K, rehydration rather than heating is required to restore structural diffusion. At λ = 14, nine of twenty-four trajectories sustain structural diffusion (2.1–3.1 hops/ps) while the rest stay kinetically locked; velocity-reseed controls show this identity is configuration-determined. Individual hops displace the excess charge by only ≈0.4 Å, so net transport relies on the minority of cage-escaping hops, implying Haven-ratio-type corrections of up to ≈10× on the tracer-based conductivities. Two distinct mechanistic regimes are thus resolved, separated by a crossover in 6 < λ ≤ 14; density-related uncertainty chiefly bounds the quantitative rates at 230 K.

## 1. Introduction

Proton exchange membrane fuel cells (PEMFCs) represent one of the most promising electrochemical energy conversion technologies for automotive and stationary power applications, owing to their high energy conversion efficiency, rapid start-up, and zero tailpipe emissions [1]. The perfluorosulfonic acid (PFSA) ionomer membrane, commercially exemplified by Nafion^®^, remains the electrolyte of choice because of its high proton conductivity under hydrated conditions, chemical stability, and mechanical robustness. Proton transport through this membrane governs the ohmic resistance of the cell and thereby directly limits achievable power density. Understanding the microscopic mechanism of proton conduction in PFSA membranes is therefore a prerequisite for rational membrane design and performance optimization, particularly under the reduced hydration and sub-ambient temperatures encountered during cold-start operation [1,2]. Cold-start success or failure hinges on the transport and phase change of supercooled water through the membrane, catalyst layer, and gas diffusion layer [3], and repeated freezing starts cause irreversible degradation of the membrane electrode assembly [4]. Experiments further show that proton conduction can be sustained well below 0 °C, and that structural engineering of the membrane can markedly enhance and stabilize this sub-zero conductivity [5,6].

Two limiting mechanisms have long been invoked to describe proton transport in hydrated polymer electrolytes. In the Grotthuss (structural diffusion) mechanism, the excess proton hops along a pre-existing hydrogen-bond network: the hydronium ion ($H_{3}O^{+}$) donates a proton to an adjacent water molecule via O–H bond breaking and reforming, thereby propagating the positive charge without net translational motion of the oxygen sublattice [7]. Ab initio molecular dynamics, full-dimensional quantum dynamics, and broadband two-dimensional infrared spectroscopy have since shown that the hydrated proton interconverts continuously between Zundel- and Eigen-type complexes, with proton transfer driven by coordination fluctuations of the first solvation shell [8,9,10]. In the vehicle mechanism, by contrast, the proton is carried within the diffusive motion of the hydrated proton complex itself, primarily $H_{3}O^{+}$, and its mobility is limited by the translational diffusion coefficient of the carrier molecule [11]. These two channels are not simply additive: recent analyses of concentrated acid solutions show that structural and vehicular displacements are strongly anti-correlated, so that the observed proton diffusivity falls well below the sum of the two nominal contributions [12]. The coexistence of the two mechanisms within a single conducting polymer was established earlier by ab initio molecular dynamics [13], a picture that has been the subject of continued debate [14]. Experimental and simulation studies on Nafion indicate that Grotthuss hopping dominates at high hydration levels (λ > 10; λ = *n*($H_{2}$O)/*n*(${\mathrm{SO}}_{3}$H)), where continuous water channels facilitate percolation, whereas under confinement at low λ those studies attribute the transport to the vehicle mechanism [15,16]; Section 3.3 shows that this attribution does not survive an explicit event census at λ = 6. However, the hydration and temperature conditions under which the crossover between these two mechanisms occurs remain poorly resolved.

Classical molecular dynamics (MD) simulations have provided extensive insight into the nanostructure and water dynamics of hydrated PFSA membranes [15,17,18,19,20,21]. These studies established the morphological evolution from isolated water clusters at λ ≈ 3–5 to percolated channels at λ > 14 and quantified water and hydronium diffusion coefficients as a function of hydration. Nevertheless, classical force fields treat atoms as fixed-charge particles with prescribed bonding topologies; they cannot describe the bond-breaking and bond-forming events that constitute the elementary step of Grotthuss proton transfer. Reactive force field (ReaxFF) approaches overcome this limitation by allowing dynamic bond order evolution [22]; dedicated ReaxFF water force fields capture proton transfer in bulk water and reproduce the essential hydrogen-bond-network dynamics [23]. Recent reactive simulations that treat proton hopping explicitly reproduce Grotthuss transfer in hydrogen-bonded aqueous environments [24]. More recently, Mabuchi and Tokumasu employed a reactive molecular dynamics model (anharmonic two-state empirical valence bond) to relate proton transport directly to PFSA morphology, revealing hydration-dependent transitions in hydronium dynamics [25], while complementary MS-EVB, ab initio, and classical molecular dynamics studies have characterized the solvation, structure, and transport of excess protons in hydrated Nafion and related hydrated-proton systems [26,27,28,29,30]. ReaxFF studies have also characterized the hydration-dependent morphology and proton exchange site equilibria of fuel cell ionomers, including the protonation states of Nafion side chains [31,32], as well as the oxidative degradation pathways of PFSA membranes [33]. Most recently, Hu et al. characterized the intermittent cage-rattling and cage-jumping dynamics of hydrated protons in Nafion under applied electric fields, showing that the local chemical environment of the proton-coordinating cage governs its mobility [34]. That study employs classical molecular dynamics with a non-dissociative hydronium model, so O–H bond cleavage—the elementary step of the Grotthuss mechanism—cannot occur in it by construction; the authors identify this as a limitation and note that ab initio molecular dynamics, while appropriate to Grotthuss transfer, is too short and too small for comprehensive cage-effect statistics [34], the Grotthuss-side evidence available to them being a first-principles study of back-and-forth proton motion at low hydration [35]. Machine-learned interatomic potentials have recently begun to address proton transport in hydrated PFSA ionomers at several hydration levels [36], but reactive force fields remain the practical route to nanosecond-scale, statistically replicated reactive trajectories over the hydration and temperature matrix considered here. However, no prior study has systematically mapped the Grotthuss-to-vehicle crossover across a matrix of hydration levels and temperatures with explicit proton-transfer event statistics in a reactive force field, which is what permits the event-level census reported here. Relative to that prior work the present study contributes four things: a mechanism assignment obtained by counting bond-breaking events rather than inferred from diffusivity contrasts that are shown here to fail; a bimodal stably active/locked landscape at λ = 14 whose configuration-determined character is established by same-configuration velocity-reseed controls; a quantitative resolution of the two-order-of-magnitude discrepancy between hop rates and tracer diffusivities, via back-hop statistics and per-hop charge-centre displacements; and a temperature axis extending to 230 K with the arrested branch supported by nanosecond-scale extended sampling.

In this work, ReaxFF-MD simulations are employed to decouple the effects of hydration level and temperature on the proton transport mechanism under conditions relevant to PEMFC cold-start operation. A dataset of 48 independent trajectories is constructed, spanning four temperatures (230, 250, 270, and 300 K), two hydration levels (λ = 6 and λ = 14), and six statistical seeds, each comprising 4 million steps (≈1 ns) at a 0.25 fs timestep. The transport mechanism is diagnosed through direct identification of proton hopping events via minimum-image-convention distance matrices and time-resolved host-oxygen tracking. Relative to prior classical MD studies, which inferred the mechanism indirectly from differences between $D_{H^{+}}$ and $D_{O}$ [15,18], and to reactive simulations that addressed morphology–transport coupling without explicit event counting [25], the present work provides, with replicated trajectories, a direct event-level quantification of the Grotthuss-to-vehicle crossover across the hydration–temperature parameter space, in the incipient, pre-freezing regime of cold-start operation; ice formation, phase transitions, and catalyst-layer processes are outside the scope of this study. Because membrane-level transport emerges from strongly coupled multi-scale processes, the atomistic parameters obtained here are intended as inputs to continuum PEMFC transport and cold-start models [37,38].

## 2. Computational Methods

### 2.1. Reactive Force Field

All reactive MD simulations were performed with the ReaxFF engine implemented in AMS 2026 (SCM, Amsterdam, The Netherlands). The total potential energy is decomposed as

(1) $$E_{\mathrm{sys}}=E_{\mathrm{bond}}+E_{\mathrm{over}}+E_{\mathrm{under}}+E_{\mathrm{lp}}+E_{\mathrm{val}}+E_{\mathrm{pen}}+E_{\mathrm{tor}}+E_{\mathrm{conj}}+E_{\mathrm{vdW}}+E_{\mathrm{Coulomb}}$$

Bond orders are computed continuously from interatomic distances, incorporating σ, π, and double-π contributions:

(2) $$BO_{ij}^{\prime }=\exp\left[p_{\mathrm{bo}1}{\left(r_{ij}/r_{0}\right)}^{p_{\mathrm{bo}2}}\right]+\exp\left[p_{\mathrm{bo}3}{\left(r_{ij}/r_{0}\right)}^{p_{\mathrm{bo}4}}\right]+\exp\left[p_{\mathrm{bo}5}{\left(r_{ij}/r_{0}\right)}^{p_{\mathrm{bo}6}}\right]$$

and are further corrected by coordination functions to account for over- and under-coordination [22]:

(3) $$BO_{ij}=\frac{1}{2}\left[BO_{ij}^{\prime }+BO_{ji}^{\prime }\right]\cdot f_{1}\cdot f_{4}\cdot f_{5}$$

The corresponding bond energy (Equation (4)) thus varies smoothly with interatomic distance, describing bond formation and cleavage without discontinuous potential surfaces:

(4) $$E_{\mathrm{bond}}=-D_{e}^{\sigma }BO_{ij}^{\sigma }-D_{e}^{\pi }BO_{ij}^{\pi }-D_{e}^{\pi \pi }BO_{ij}^{\pi \pi }$$

Atomic charges are equilibrated at each step by a charge-equilibration scheme of the QEq type [39]. The force field used here (ReaxFF_PEMFC) covers six elements (C, H, O, N, S, F) and was developed alongside the PFSA degradation study of Qi et al. [33]; its applicability to the C–H–O–S–F chemistry studied here, including the proton-transfer reaction channels, is inherited from that lineage.

### 2.2. Model Construction

The PFSA membrane model was constructed with the AMS molecular modeling environment. Ten identical polymer chains were placed in a cubic periodic cell of 30 × 30 × 30 Å^3^. Each chain consists of a 24-carbon perfluorinated backbone terminated by ${\mathrm{CF}}_{3}$ groups, ${\mathrm{CF}}_{3}$–(${\mathrm{CF}}_{2}$)_4_–CH(–O–CF(${\mathrm{CF}}_{3}$)–${\mathrm{CF}}_{2}$–${\mathrm{CF}}_{2}$–${\mathrm{SO}}_{3}$H)–(${\mathrm{CF}}_{2}$)_17_–${\mathrm{CF}}_{3}$, with a single perfluorosulfonic acid side chain grafted at an inner backbone carbon (the grafting carbon carries one H). Each chain comprises 28 C, 56 F, 4 O, 1 S, and 2 H atoms; the sulfonic acid terminus is protonated (${\mathrm{SO}}_{3}$H). The equivalent weight of this architecture is EW ≈ 1498 g/mol per sulfonate group, somewhat higher than commercial Nafion (≈1100 g/mol); the higher EW reduces the volumetric density of sulfonate groups and, at a given λ, increases the water-to-sulfonate volume ratio of the water pools, which may shift the hydration threshold for Grotthuss activation relative to commercial ionomers. Quantitatively, the sulfonate molar density of the model is ≈27% below that of Nafion 117 at equal mass density, so the hydration level at which a percolated hydrogen-bond network first forms is expected to lie higher in this model than in real Nafion. The λ values quoted below should therefore be read as model-internal rather than as direct Nafion equivalents, and a threshold located precisely on the model’s λ scale would not transfer directly to commercial membranes; this is one reason the mechanistic switch is discussed in terms of hydrogen-bond connectivity as well as λ (Section 3.3). The 30 Å cell is likewise too small to accommodate long-wavelength chain packing, so the model represents a homogeneous membrane interior rather than a phase-separated morphology; this bounds the structural realism of the absolute coefficients reported below.

Two hydration levels were prepared: λ = 6 (60 $H_{2}$O) and λ = 14 (140 $H_{2}$O), where λ = *n*($H_{2}$O)/*n*(${\mathrm{SO}}_{3}$H). The λ = 6 system comprises 1090 atoms (280 C, 560 F, 140 H, 100 O, 10 S) and the λ = 14 system 1330 atoms (280 C, 560 F, 300 H, 180 O, 10 S), corresponding to initial densities of ≈0.99 and ≈1.08 ${g/\mathrm{cm}}^{3}$. Model parameters are summarized in Table 1.

O–H-bound hydrogens: 130 at λ = 6 (120 water H + 10 sulfonic-acid H) and 290 at λ = 14 (280 water H + 10 sulfonic-acid H); the ten sulfonic-acid protons are the net charge carriers ($n_{\mathrm{carrier}}$).

### 2.3. Simulation Protocol

Each system was prepared with a three-stage protocol: (1) **Geometry optimization** (conjugate gradients, maximum 500 iterations, gradient convergence 0.01 hartree/Å) was performed with the NonReactive constraint to remove atomic overlaps. (2) **Heating/equilibration** was performed in the NVT ensemble: the system was initialized at 10 K with random velocities and heated to the target temperature over 25 ps (100,000 steps of 0.25 fs) using the Berendsen thermostat with a coupling constant τ = 50 fs, again with the NonReactive constraint to prevent premature bond-breaking. Because the Berendsen scheme suppresses rather than reproduces canonical temperature fluctuations, it is used solely for thermal control, and no equilibrium thermodynamic averages are extracted from the thermostat dynamics. (3) **Production**: The NonReactive constraint was removed and each configuration (six independent seeds per condition) was propagated for 4,000,000 steps (1 ns) at a timestep of 0.25 fs under NVT conditions with the Nosé–Hoover chain (NHC) thermostat (τ = 100 fs). All three stages were performed at constant volume: no barostat was applied at any stage, so the cell retained its as-built 30 × 30 × 30 Å^3^ dimensions throughout, and the mass density remained at its initial value (≈0.99 ${g/\mathrm{cm}}^{3}$ at λ = 6; ≈1.08 ${g/\mathrm{cm}}^{3}$ at λ = 14). The implications of this fixed-cell protocol are assessed in Section 3.4.

Trajectory frames were saved every 200 steps (50 fs), yielding ≈20,000 frames per trajectory. The Grotthuss hop census and excess-charge-center (CEC) analyses used all saved frames at their native 50 fs resolution, whereas the MSD-fitting and RDF analyses subsampled every fourth saved frame (200 fs effective resolution). Checkpoints were written every 5000 steps. A total of 48 independent production runs were performed: 4 temperatures (230, 250, 270, 300 K) × 2 hydration levels (λ = 6, 14) × 6 seeds.

### 2.4. Analysis Methods

**Diffusion coefficients and conductivity:** Three transport quantities are distinguished throughout and should not be conflated: (i) the tracer (self-)diffusion coefficient $D_{H^{+}}$ of the ten initially acidic protons, followed by atom index; (ii) the excess-charge-center diffusion coefficient $D_{\mathrm{CEC}}$, which tracks the diffusing charge defect itself (defined below); and (iii) their ratio $H_{R}=D_{\mathrm{CEC}}/D_{H^{+}}$, a Haven-ratio-type correlation correction. $D_{H^{+}}$ equals the charge-carrier displacement only in the absence of Grotthuss exchange; once hopping occurs, $D_{\mathrm{CEC}}$ is the physically meaningful transport coefficient, and the Nernst–Einstein conductivity of Equation (6), which is built on $D_{H^{+}}$, must be read as a tracer-based estimate subject to the $H_{R}$ correction (Section 3.3). The ten charge-carrying protons were identified at the first trajectory frame as the hydrogen atoms covalently bound to the sulfonate hydroxyl oxygens of the ten ${\mathrm{SO}}_{3}$H groups (O–H distance < 1.30 Å) and were tracked by atom index through the entire trajectory. Mean-square displacements (MSD) were computed for these tracer protons and for all oxygen atoms. Because the tracers are followed by atom index, an acidic proton that transfers away from its parent sulfonate continues to be tracked as a water proton; $D_{H^{+}}$ is therefore the tracer (self-)diffusion coefficient of the initially acidic protons, not a charge-carrier displacement in Grotthuss-active trajectories (Section 3.3). The MSD definition used throughout is shown in Equation (5):
  (5) $$\mathrm{MSD}\left(t\right)=\langle |r\left(t\right)-r\left(0\right){|}^{2}\rangle$$

The diffusion coefficient *D* was extracted from the linear regime of MSD(*t*) = 6Dt via least-squares fitting over a per-trajectory window selected automatically as the interval maximizing the fit $R^{2}$ among ten candidate windows spanning 5–400 ps ((5, 50), (10, 100), (20, 100), (10, 200), (20, 200), (50, 200), (50, 300), (100, 300), (100, 400), (50, 400) ps). Because $R^{2}$-maximization alone can favor a locally linear but non-diffusive segment, the selection was cross-checked with a physical criterion based on the local log–log slope of the MSD: a Fickian window was defined as the longest contiguous lag range over which α(t)=dlnMSD/dlnt remains within [0.8, 1.2]. The outcome of this two-step selection, namely the fraction of trajectories exhibiting a Fickian regime and the resulting sensitivity of the seed-averaged diffusion coefficients, is assessed in Section 3.1. Proton conductivity was derived from the Nernst–Einstein relation (Equation (6)):

(6) $$\sigma =\frac{nq^{2}D}{k_{B}T}$$

where *n* = 10/$V_{\mathrm{cell}}$ is the number density of the charge-carrying sulfonic-acid protons (Table 1). The cell contains 130 O–H-bound hydrogens at λ = 6 and 290 at λ = 14; only the ten sulfonic-acid protons are net charge carriers. A sensitivity analysis that tracks all 130 (290) O–H-bound hydrogens instead of the ten tracer protons raises $D_{H^{+}}$ by a factor of ∼1.3–2 and, if all O–H-bound hydrogens were additionally counted as carriers, would raise σ by one to two orders of magnitude; because water protons carry no net charge in the absence of Grotthuss exchange, the ten-proton tracer population is used throughout.

**Grotthuss hopping detection:** At each saved trajectory frame (50 fs native resolution), every hydrogen was first assigned to its nearest oxygen within a cutoff of 1.30 Å under the minimum image convention; the “host” oxygen carrying the excess charge was then taken as the single oxygen with the largest number of assigned hydrogens in that frame. A Grotthuss hop event was registered when this host oxygen index changed between consecutive saved frames. Three consequences of this single-centre construction are stated here because they affect the interpretation of the rates reported below. First, the hop counts and hop rates refer to the host changes of one dominant excess-charge centre per cell per frame, not to the summed activity of the ten acidic protons; the values of 2.1–3.1 hops/ps quoted at λ = 14 are therefore per-defect rates. Second, when more than one hydronium-like defect coexists in a frame, the argmax selects one of them and the others are not tracked in that frame. Third, ties in the assigned-hydrogen count are broken by the lower oxygen index, and a change of host between two tied oxygens is registered as a hop. Requiring at least three assigned hydrogens on the host in both frames and a host–host separation within 3.5 Å reduces the total census from 21,572 to 8366 events. This construction is justified by the measured defect population: the stably active λ = 14 trajectories sustain a mean of only 1.1–1.3 $H_{3}O^{+}$-like defects per frame (Section 3.3), so the dominant centre accounts for the excess charge in the large majority of frames. The total hop count, hop rate (hops/ps), and per-window distribution (250 ps bins) were tabulated. To quantify the sensitivity of the census to analysis cadence, the 50 fs series was additionally coarse-grained to 200 fs (every fourth frame) to reproduce the cadence of the originally reported counts; for each trajectory the fraction of 50 fs events missed by the 200 fs sampling (miss rate) and the fraction of hops returning to the previous host within 200 fs (back-hop fraction) were computed. To quantify the sensitivity of the census to the covalent O–H cutoff, the carrier identification and the CEC centroid construction were repeated at cutoffs of 1.25, 1.30, 1.35, and 1.40 Å: on all 48 trajectories the ten-carrier set is identical at every cutoff (Jaccard index 1.0), and the CEC centroid shifts by less than 0.0001 Å, confirming that the 1.30 Å criterion is robust within ±0.05 Å; the host-oxygen assignment itself is cutoff-free by construction (nearest-oxygen assignment).
**Excess-charge-center (CEC) analysis:** To separate tracer (self-)diffusion from genuine charge displacement, the excess-charge center of the hydrated proton was tracked at the native 50 fs resolution on all trajectories, following the multistate empirical valence bond (MS-EVB) definition of the proton charge centroid [40,41]. At each frame, the host oxygen $O^{*}$ (the oxygen with the largest number of assigned hydrogens, as in the hop census) and its covalently bound hydrogens $H_{i}$ (*r* < 1.30 Å) define the CEC position $r_{\mathrm{CEC}}=r_{O^{*}}+\sum_{i}w_{i}\left(r_{H_{i}}-r_{O^{*}}\right)$, with inverse-distance weights $w_{i}$ normalized to unity. The CEC mean-square displacement yields a charge-diffusion coefficient $D_{\mathrm{CEC}}$ directly comparable with $D_{H^{+}}$. The ratio $H_{R}=D_{\mathrm{CEC}}/D_{H^{+}}$ plays the role of a Haven ratio: it quantifies the correlation correction inherent in Nernst–Einstein conductivities derived from tracer diffusion, which is exact only when charge transport is purely vehicular [42,43]. Because the charge centre is constructed from the same single argmax host as the hop census, $D_{\mathrm{CEC}}$ is the diffusion coefficient of one dominant charge defect, whereas $D_{H^{+}}$ is averaged over the ten indexed tracer protons. $H_{R}$ therefore compares a single-defect charge diffusion with a ten-proton tracer average and is not a Haven ratio in the strict single-particle sense; it is used throughout as an order-of-magnitude correlation correction, and the extreme per-seed values reported in Section 3.3 are quoted as a range bound only. The carrier density *n* = 10/$V_{\mathrm{cell}}$ remains the appropriate choice under Grotthuss transport, since there is still one excess-charge defect per sulfonate group; what changes is which particle carries the defect, not how many defects exist. In addition, the per-hop CEC displacement $|\Delta r_{\mathrm{CEC}}|$ (the CEC displacement across a host-change event) was recorded for every registered hop.
**Radial distribution functions:** O–O and O–H pair correlation functions g(r) were computed between water-type oxygens ($O_{w}$: oxygens carrying ≥2 covalently bound hydrogens within 1.30 Å, i.e., $H_{2}$O and $H_{3}O^{+}$; polymer ether and sulfonate oxygens excluded) and their associated hydrogens, from trajectory frames using standard histogram methods with a bin width of 0.02 Å (Equation (7)):
  (7) $$g\left(r\right)=\frac{\langle n(r)\rangle }{4\pi r^{2}\Delta r\cdot \rho_{0}}$$

Running coordination numbers (CN) were obtained by numerical integration of 4$\pi \rho r^{2}g\left(r\right)$; first-shell values are reported to the first minimum beyond the main peak.

**Pure-water benchmark of the proton-transfer barrier:** To anchor the event-level statistics and the proton-transfer barrier implicit in the force field against known physics, the excess-proton diffusion benchmark of bulk water [44] was reproduced with the identical ReaxFF_PEMFC potential: a periodic cell of 256 $H_{2}$O molecules plus one excess proton ($H_{3}O^{+}$, 769 atoms) was equilibrated at 300 K and simulated for 100 ps in each of four independent seeds (NVT, 0.25 fs time step, 50 fs frame interval), followed by the same hopping census, CEC construction, and MSD analysis as applied to the membrane systems (benchmarked in Section 3.3).
**Colour convention for all figures:** Two colour roles are used throughout and should not be conflated. In panels that classify trajectories by mechanism, red denotes stably active (Grotthuss-hopping) trajectories and grey denotes locked trajectories. In panels that compare hydration levels, blue denotes λ = 6 and red denotes λ = 14. Temperature-resolved panels use a single-hue sequential scale, light to dark for 230 to 300 K. The overall mnemonic is red = conducting/high-hydration/warm and grey–blue = locked/low-hydration/cold; red/green combinations are avoided for colour-vision accessibility.
**Arrhenius analysis:** Activation energies were determined from linear fits of ln(*k*) versus 1000/*T* across the four temperatures (Equation (8)):
  (8) $$\ln k=\ln A-\frac{E_{a}}{k_{B}T}$$
  where *k* is the seed-averaged conductivity (or diffusion coefficient), yielding $E_{a}$ in eV directly from the slope ($-E_{a}/k_{B}$ × $10^{-3}$). Uncertainties were estimated by bootstrap resampling of the six seeds (2000 replicates; Section 3.1).

## 3. Results and Discussion

### 3.1. Proton Diffusion, Conductivity, and Arrhenius Kinetics

**Figure 1:**
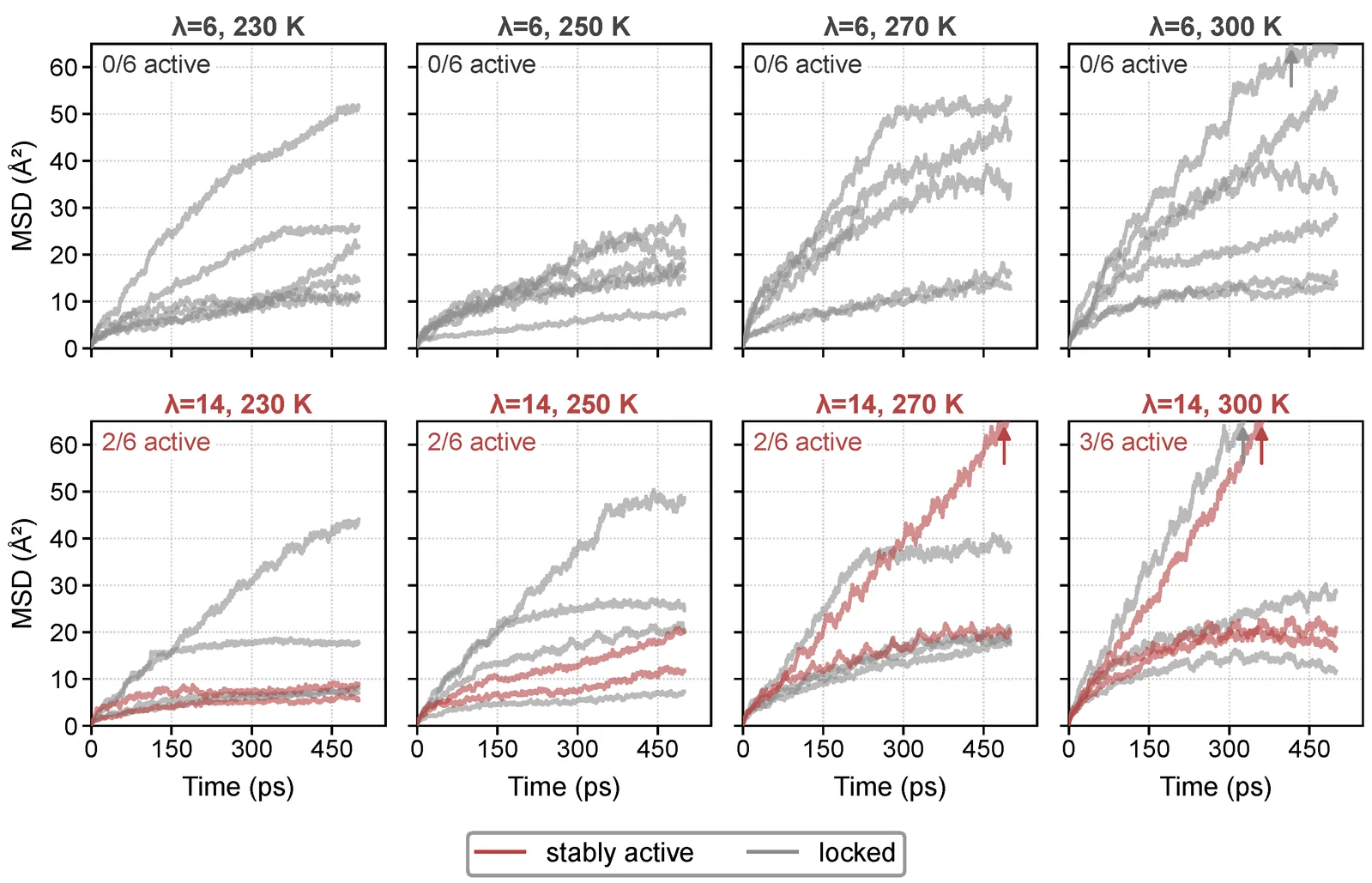
Mean-square displacement of the ten sulfonic-acid tracer protons over the first 500 ps of each trajectory, shown per seed rather than as a seed mean, at 230–300 K for λ = 6 (**top row**) and λ = 14 (**bottom row**). Each panel draws the six individual seed curves coloured by trajectory class from the hop census of Section 3.3, following the paper-wide mechanism-colour convention (red = stably active, grey = locked) at a common line weight and partial opacity (α ≈ 0.6), so that overlapping seeds read as a density band; the legend is placed below the panels. No mean or dispersion band is drawn, so that the per-seed spread—the quantity underlying the limited number of Fickian windows discussed in Section 3.1—is visible. Because the two classes are distinguished by a red/grey contrast rather than by two saturated hues, they remain separable in greyscale, and the class of every curve is in addition recoverable from the per-panel active count given in each panel corner. The ordinate is truncated at 65 Å^2^ so that the 44 curves lying below it fill the panel; the four that do not (66.1–93.8 Å^2^) are marked by an upward arrow, drawn in the colour of the corresponding curve, at the point where they leave the plotted range. Note that the tracer MSD does not separate the two classes: a locked trajectory, in which the acidic proton retains a fixed host oxygen, can still exhibit large tracer MSD through vehicular motion of its host water molecule. At λ = 14, 300 K the two largest displacements in the entire dataset belong to one locked seed (93.8 Å^2^) and the most mobile stably active seed (90.1 Å^2^). The mechanism is therefore diagnosed by the hop census, and $D_{H^{+}}$ is read as a tracer (self-)diffusion coefficient. The tracer diffusion coefficient is extracted from the per-trajectory linear-regime window selected by maximizing the fit $R^{2}$ (Section 2.4).

**Figure 2:**
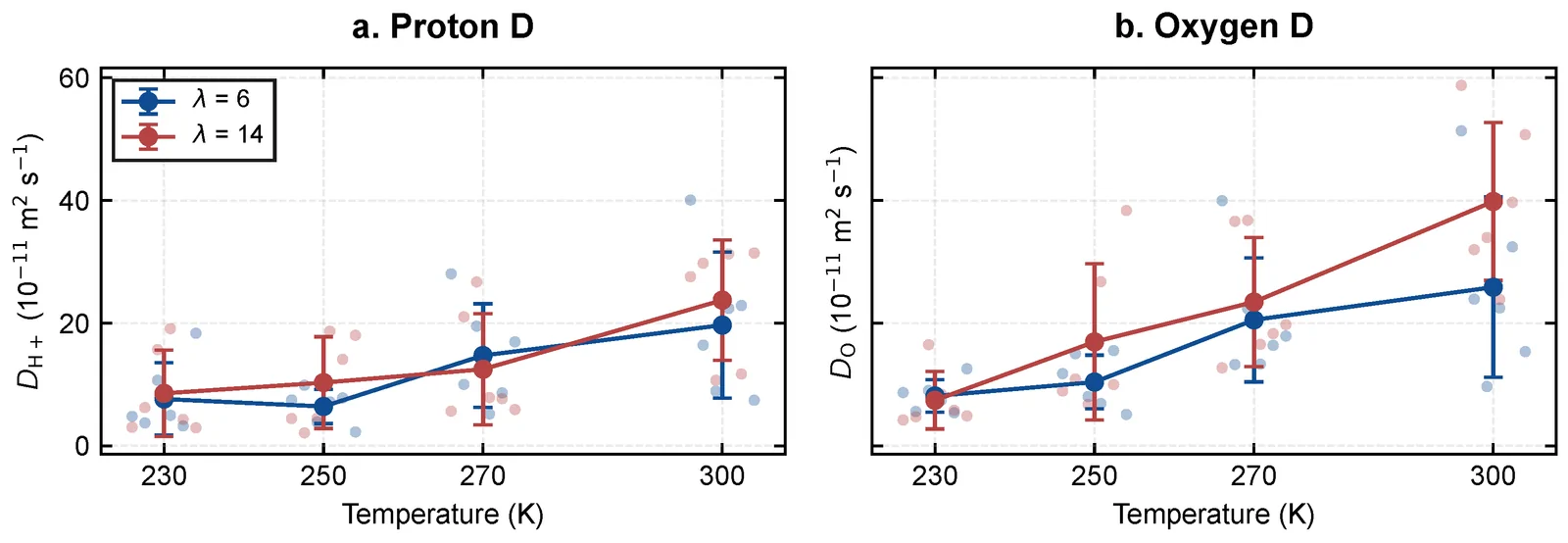
Diffusion coefficients of the (**a**) sulfonic-acid tracer protons and (**b**) oxygen atoms versus temperature; faint dots = individual seeds, bold markers = mean ± s.d.

**Figure 3:**
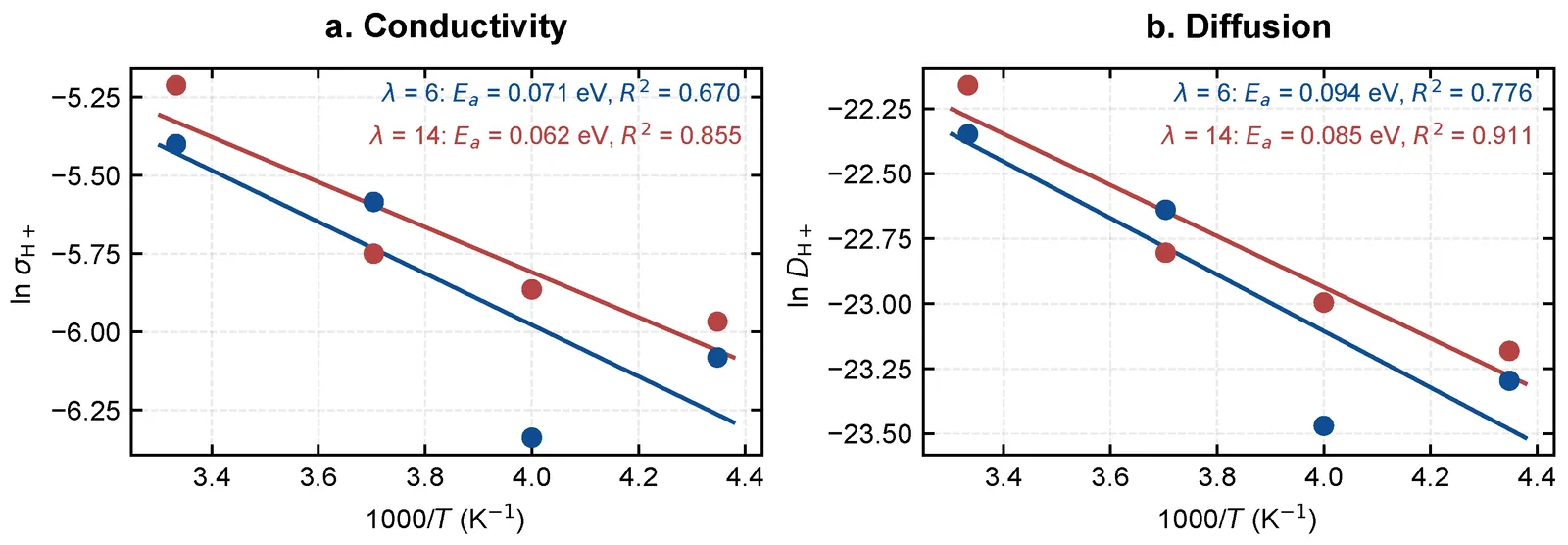
Arrhenius plots of (**a**) proton conductivity and (**b**) proton diffusion coefficient with linear fits; $E_{a}$ and $R^{2}$ annotated per hydration level.

**Figure 4:**
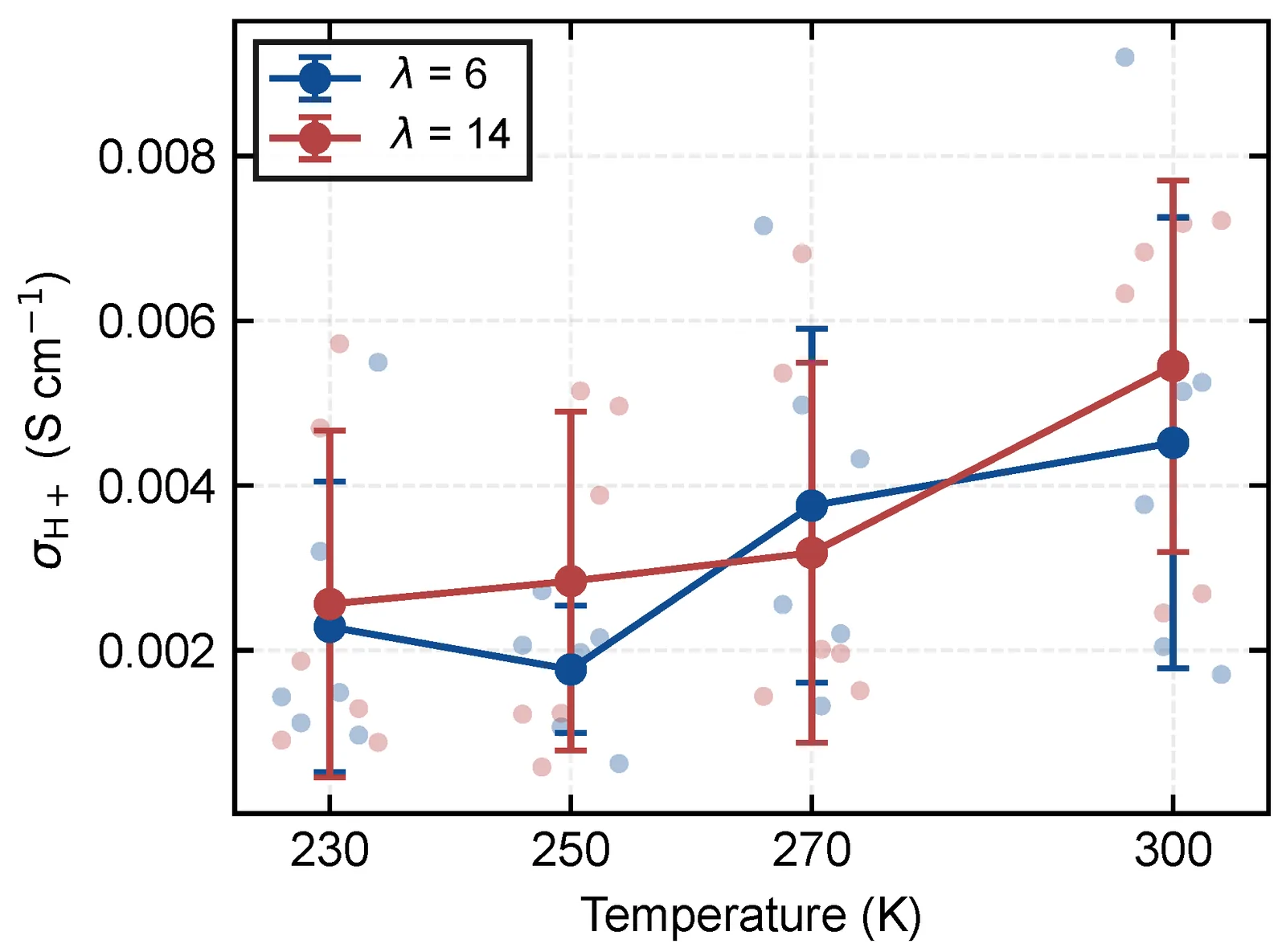
Proton conductivity versus temperature; faint dots = individual seeds, bold markers = mean ± s.d.

Proton transport was characterized from the mean-square displacement (MSD) of the ten charge-carrying sulfonic-acid protons ($D_{H^{+}}$) and of all oxygen atoms ($D_{O}$), averaged over six independent seeds per condition, from which the Nernst–Einstein conductivity (Equation (6)) was in turn derived; Figure 1, Figure 2, Figure 3 and Figure 4 and Table 2 summarize the results. The tracer-proton MSD curves (Figure 1) are sub-diffusive over most of the accessible lag range: applying the log–log slope criterion of Section 2.4, only 10 of the 48 trajectories reach a Fickian window (α(t) within [0.8, 1.2], typically 2–35 ps long) inside the 1 ns observation window, whereas the remaining 38 stay sub-diffusive (α<0.8) throughout, evident as the sub-linear plateaus in Figure 1. The automatic $R^{2}$-maximizing window selection therefore carries a per-trajectory sensitivity: individual-seed *D* values span a median factor of ∼3 (90th percentile ≈7) across the candidate windows, with the single outlier trajectory (λ = 14, 230 K; termed marginal in Section 3.3) reaching ≈70×, and the seed-averaged *D* values obtained with the physical Fickian windows exceed the $R^{2}$-selected values by factors of 3–13. The tabulated coefficients are consequently order-of-magnitude effective diffusivities rather than converged material constants, with the per-trajectory scatter averaged over in the six-seed statistics.

Extracted from the linear MSD regime, $D_{H^{+}}$ at λ = 6 rises from (6.4–7.6) × $10^{-11}\;m^{2}$/s at 230–250 K to (19.7 ± 11.9) × $10^{-11}\;m^{2}$/s at 300 K, and at λ = 14 from (8.6 ± 7.0) × $10^{-11}\;m^{2}$/s at 230 K to (23.7 ± 9.8) × $10^{-11}\;m^{2}$/s at 300 K (Figure 2a). The tracer statistics carry substantial seed-to-seed scatter (s.d. ≈ 40–80% of the mean), reflecting the small number (ten) of tracked protons. As Figure 2b shows, $D_{O}$ is comparable in magnitude to $D_{H^{+}}$ across all conditions and the two coefficients cannot be told apart within their seed-to-seed scatter; this is precisely the regime in which the conventional $D_{H^{+}}$–$D_{O}$ contrast loses its diagnostic power as a mechanism proxy, and it motivates the direct event-level census developed in Section 3.3.

The conductivity-based Arrhenius fits (Figure 3a) give activation energies of $E_{a}$ = 0.071 eV ($R^{2}$ = 0.67) for λ = 6 and 0.062 eV ($R^{2}$ = 0.86) for λ = 14, while the corresponding fits of the tracer diffusion coefficients (ln $D_{H^{+}}$ vs. 1000/*T*; Figure 3b) yield 0.094 eV ($R^{2}$ = 0.78) and 0.085 eV ($R^{2}$ = 0.91); the four fits thus span 0.062–0.094 eV, i.e., $E_{a}$ = 0.06–0.09 eV to the precision warranted here. The ≈0.02 eV offset between the two quantities is the expected $k_{B}T$ factor from σ ∝ D/T. These barriers fall at the lower edge of experimental measurements on liquid-water-equilibrated Nafion 117 (0.09–0.16 eV) [45,46]. Bootstrap resampling of the six seeds (2000 replicates) gives 95% confidence intervals of 0.037–0.152 eV (λ = 6) and 0.035–0.149 eV (λ = 14) for the diffusion-based barriers; the substantial overlap indicates that the two are not statistically distinguishable with the present sampling, so mechanistic contrasts between hydration levels should not be inferred from $E_{a}$ alone. The lower $R^{2}$ values obtained for λ = 6 (0.78 for diffusion, 0.67 for conductivity) reflect the substantial seed-to-seed scatter of the intermittent, localized acidic-proton motion combined with the four-point fit, so the λ = 6 barriers are apparent activation energies and upper bounds. These confidence intervals span a factor of ≈4, overlap each other, and overlap the entire experimental range of 0.09–0.16 eV [45,46], so the activation energies reported here are order-of-magnitude anchors rather than material constants. Because only 10 of the 48 trajectories reach a Fickian regime, the mechanistic conclusions rest on the event-level census of Section 3.3, which does not depend on MSD window selection.

The Nernst–Einstein conductivities (Figure 4) track the tracer diffusivities: at λ = 6 they rise from (1.8–2.3) × $10^{-3}$ S/cm at 230–250 K to (4.5 ± 2.7) × $10^{-3}$ S/cm at 300 K, and at λ = 14 from (2.6 ± 2.1) × $10^{-3}$ to (5.5 ± 2.3) × $10^{-3}$ S/cm. Because the carrier population is identical at both hydration levels (ten sulfonic-acid protons per cell), the modestly higher conductivity at λ = 14 arises from the higher proton mobility in the more continuous water channels. These absolute values lie roughly an order of magnitude below experimental Nafion conductivities. The gap can be decomposed into contributions that are artefacts of the model or of the extraction procedure rather than of the transport physics. (i) Carrier density: The model carries *n* = 10/(30 Å)^3^ = 3.70 × $10^{20}\;{\mathrm{cm}}^{-3}$, whereas Nafion 117 at λ = 14 carries 7.45 × $10^{20}\;{\mathrm{cm}}^{-3}$ (1 mol ${\mathrm{SO}}_{3}$H per 1100 g polymer; dry volume 555.6 ${\mathrm{cm}}^{3}$ at ρ = 1.98 ${g/\mathrm{cm}}^{3}$, plus $252.2\;{\mathrm{cm}}^{3}$ of water for 14 × 18.015 g [47]). Since σ∝n, this factor of 2.01 is attributable entirely to the higher equivalent weight and the under-dense fixed cell. The model is internally consistent in this respect: ten chains of 1498.3 g/mol plus 140 waters give 2.907 × $10^{-20}$ g in 2.7 × $10^{-20}\;{\mathrm{cm}}^{3}$, i.e., 1.077 ${g/\mathrm{cm}}^{3}$, matching the ≈1.08 ${g/\mathrm{cm}}^{3}$ of Section 3.4. (ii) Tracer versus charge diffusion: Where Grotthuss hopping operates, the Nernst–Einstein relation built on $D_{H^{+}}$ underestimates charge transport, the physically meaningful coefficient being $D_{\mathrm{CEC}}$; on the stably active λ = 14 subset $H_{R}$ = 3–55 on condition means, implying an upward correction of ≈3–10× (Section 3.3). (iii) Window sensitivity: $D_{H^{+}}$ is extracted from sub-diffusive MSD curves over 2–35 ps windows and carries a factor 3–13 sensitivity to that choice (above). Applying (i) together with the lower end of (ii) to the λ = 14, 300 K value of 5.5 × $10^{-3}$ S/cm gives $\sigma_{\mathrm{CEC}}\approx$ 0.03–0.11 S/cm, which brackets the experimental range at comparable hydration. The discrepancy is therefore accounted for by the carrier density of the model and by the tracer-versus-charge distinction, not by a failure of the transport physics. The same decomposition shows why the λ = 6 values cannot be compared with experiment at all: no long-range charge transport occurs there (Section 3.3).

The implications for PEMFC cold-start performance are as follows. During cold-start from sub-zero temperatures, the membrane experiences reduced hydration and low temperature simultaneously. Recent investigations have characterized ice formation within the catalyst layer during cold start and its evolution with operating conditions [48], and two distinct freezing mechanisms, direct in-layer freezing at deep sub-zero temperatures and freezing of interfacial supercooled water near 0 °C, have been identified [49]. These processes occur in the catalyst layer and at interfaces that are not represented in the present membrane-interior model. Within the pre-freezing, membrane-interior regime addressed here, the present results indicate that no Grotthuss events are resolved at λ = 6 and no hydronium-like motif is detected, so proton transport, to the extent it occurs, is localized motion of the acidic proton within its sulfonate first shell together with segmental displacement of the sulfonate group itself (Section 3.3). Both are thermally activated and slow markedly on cooling, and the alternative structural-diffusion channel is absent altogether, so proton conductivity will be severely degraded. The apparent Arrhenius activation energy of 0.07 eV (conductivity-based; 0.09 eV from the diffusion fit; Section 3.1) measured here for λ = 6 quantifies this degradation and provides a molecular-level, order-of-magnitude reference for continuum cold-start models that parameterize proton conductivity at sub-zero temperatures [2,38]; its quantitative transferability is bounded by the fixed-cell density, the high equivalent weight, and the absence of ice and catalyst-layer environments (Section 3.4). Recent cold-start experiments and numerical analyses further identify the initial membrane hydration as the controlling variable of cold-start capability [50,51], so a molecular-level account of how hydration state selects the transport mechanism bears directly on this parameterization need. Two features of the present design bear on that need. First, the hydration and temperature axes are varied factorially rather than jointly, so the result is a map σ(λ,T) that a coupled continuum cold-start model can interpolate along its own simultaneous *T*–λ trajectory; a coupled molecular simulation would instead provide a single path through that map. Second, the map is strongly anisotropic: because λ = 6 shows zero resolved hopping at every temperature including 300 K, the mechanistic switch is controlled by hydration and not by temperature, so rehydration rather than heating is what restores structural diffusion. Ice formation, phase change and catalyst-layer processes remain outside the scope of a membrane-interior model (Section 3.1 and Section 3.4).

### 3.2. Solvation Structure and Radial Distribution Functions

**Figure 5:**
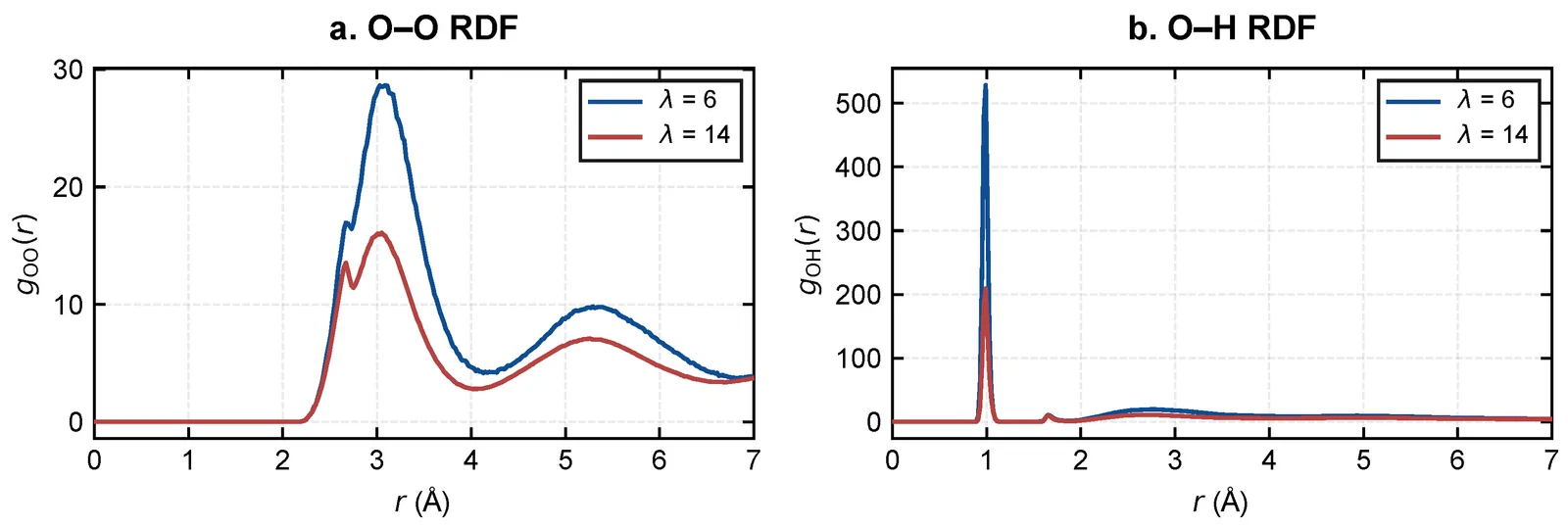
(**a**) $O_{w}$–$O_{w}$ and (**b**) $O_{w}$–$H_{w}$ radial distribution functions at λ = 6 and λ = 14 (water-type oxygens only, polymer oxygens excluded; seed-averaged over six seeds and four temperatures). At λ = 14 the split first shell of panel (**a**) reflects short, strongly hydrogen-bonded O–O contacts at ∼2.67 Å and longer hydrogen-bonded neighbors at ∼3.01–3.05 Å.

To connect these transport coefficients with the local water structure, Figure 5 presents the O–O and O–H radial distribution functions (RDFs) at λ = 6 and λ = 14.

The $O_{w}$–$O_{w}$ RDF at λ = 14 shows a split first shell with maxima at ∼2.67 Å and ∼3.01–3.05 Å (peak heights *g* ≈ 12–16, elevated by channel confinement) and a running coordination number of ∼4.5 up to the first minimum at ∼3.1 Å (300 K), close to the fourfold coordination of a tetrahedral hydrogen-bond network but with a confinement-broadened distribution. At λ = 6, the first shell is weaker and incomplete (CN ≈ 2.7 to the first minimum near 3.05 Å; ∼4.5 by 3.3 Å), reflecting the confinement of water in small clusters. The $O_{w}$–$H_{w}$ RDF (Figure 5b) complements this picture: the sharp covalent O–H peak at ∼1.0 Å and the hydrogen-bond shoulder beyond ∼1.5 Å mirror the split first shell of panel (a) and confirm that water protons remain predominantly engaged in hydrogen bonding at both hydration levels.

**Figure 6:**
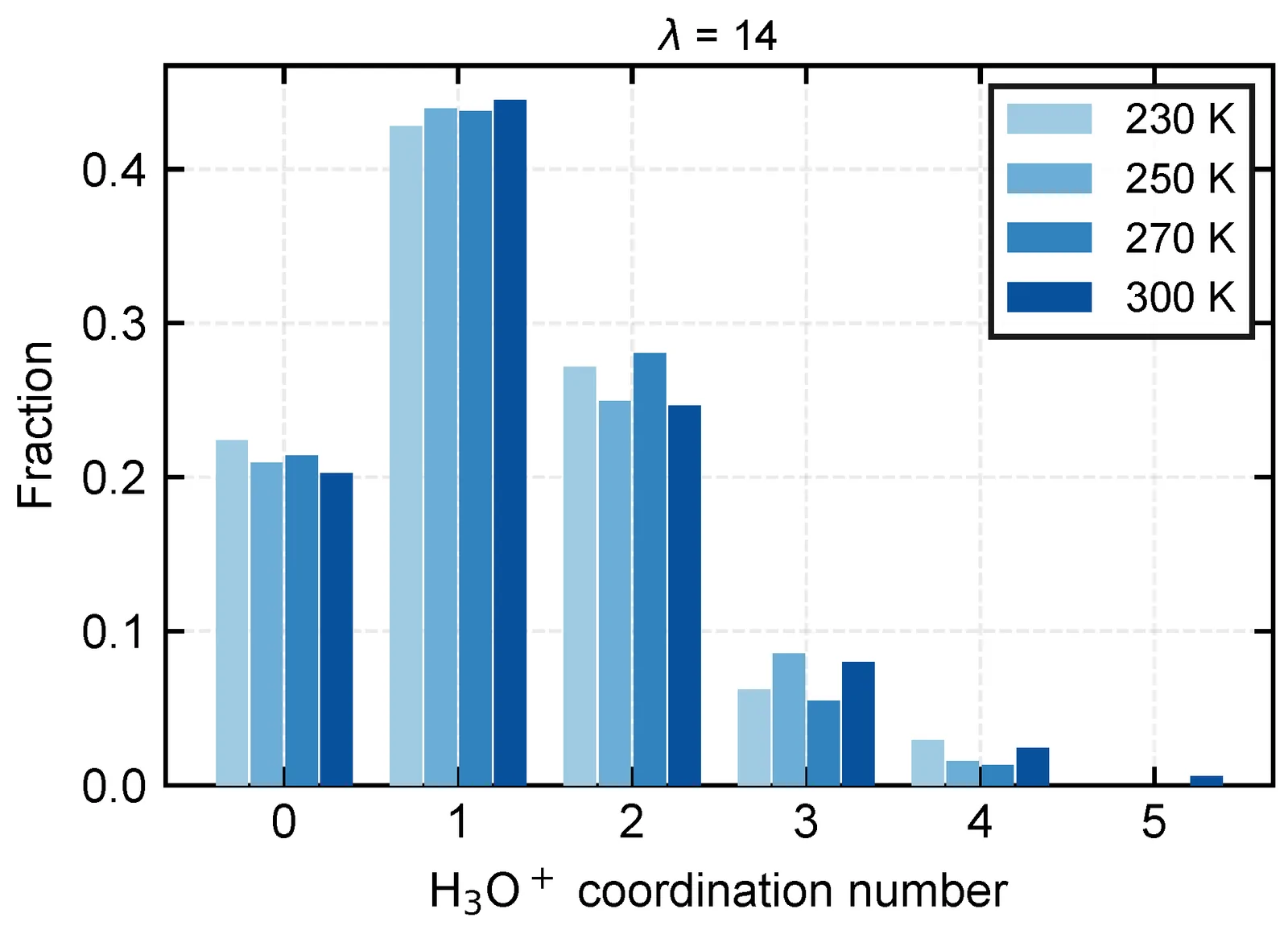
Distribution of the $H_{3}O^{+}$ coordination number at λ = 14 for the four temperatures (stably active trajectories); temperatures are coloured on a single-hue sequential scale (light → dark for 230 → 300 K) rather than a diverging blue–red scale, consistent with the colour convention of Section 2.4.

**Figure 7:**
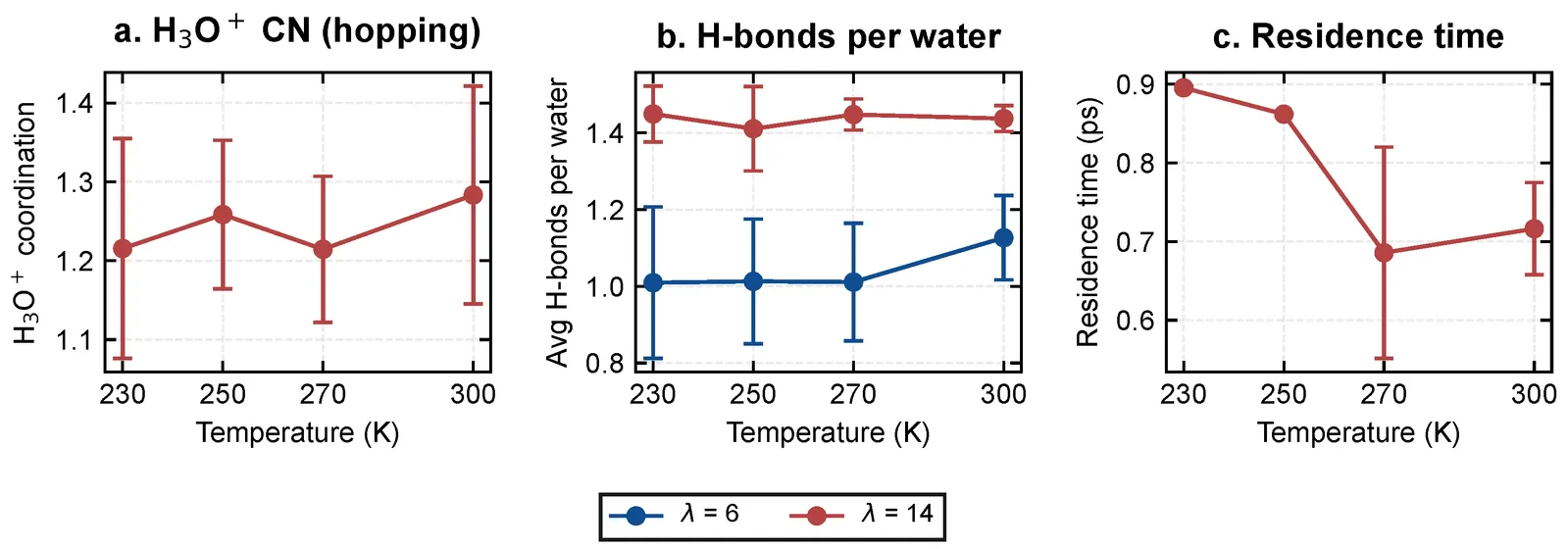
(**a**) $H_{3}O^{+}$ coordination number (stably active trajectories), (**b**) hydrogen bonds per water molecule, and (**c**) proton host-oxygen residence time versus temperature for the stably active λ = 14 trajectories; the λ = 6 systems undergo no host change and are omitted from (**c**) (residence time = full 1 ns window).

The $H_{3}O^{+}$ solvation analysis (Figure 6 and Figure 7) shows that the hydronium coordination-number distribution peaks at CN = 1 (≈44% at all temperatures; Figure 6), with a mean of 1.21–1.28 across 230–300 K (Figure 7a) for the stably active λ = 14 trajectories (the hopping-competent subset; the stably active, marginal, and locked trajectory classes are defined in Section 3.3). In addition, the CN = 0 fraction amounts to ≈20% (Figure 6): in roughly one fifth of frames the hydronium carries no hydrogen-bond acceptor at all, an isolated-defect population that is consistent with the enlarged water–water separation of the low-density fixed cell and with a hopping mechanism requiring only a single acceptor to proceed, in line with the minimal hydrogen-bond-relay picture of the Grotthuss mechanism [7]. The excess proton therefore resides predominantly as $H_{3}O^{+}$ retaining a single hydrogen-bond acceptor. Two interpretations of this under-coordination should be considered jointly. On the one hand, it is consistent with a mechanistic picture in which Grotthuss hopping requires only a single hydrogen-bond acceptor adjacent to the hydronium, rather than a fully tetrahedral solvation shell, and it agrees with the reduced hydronium coordination reported for confined and clustered hydrated-proton complexes in prior simulations of Nafion-type ionomers and model protonated-water systems [26,27,28]. On the other hand, a mean CN below two may partly reflect the reduced density of the fixed-cell model (≈1.08 ${g/\mathrm{cm}}^{3}$; Section 3.4), which enlarges the average water–water separation and lowers the expected number of hydrogen-bond acceptors. Consistent with the incomplete first coordination shell at λ = 6 evident in the RDF above (Figure 5a), the hydrogen-bond count per water molecule (Figure 7b) is considerably lower at λ = 6 (≈1.0) than at λ = 14 (≈1.4), quantifying the absence of continuous hydrogen-bond pathways, and hence of Grotthuss transport, at low hydration. The host-oxygen residence time of the excess proton on the stably active λ = 14 trajectories (Figure 7c) remains sub-picosecond (≈0.7–0.9 ps) at all four temperatures; i.e., each host oxygen retains the excess proton for only a few tenths of a picosecond before the next transfer. The corresponding contrast with the locked λ = 14 and λ = 6 trajectories, whose residence time equals the full observation window, is quantified in Section 3.3.

### 3.3. Grotthuss Hopping Statistics and Mechanism Crossover

The central result of the present work concerns the direct identification of proton transfer events. The identity of the “host” oxygen atom carrying the excess charge was tracked at every trajectory frame using a minimum-image-convention distance criterion (O–H bond length < 1.30 Å), taking the single oxygen with the largest number of assigned hydrogens in each frame (Section 2.4). A Grotthuss hop is registered when this host oxygen index changes between consecutive frames; the resulting rates are per-defect rates for the dominant charge centre, not summed over the ten acidic protons.

**Figure 8:**
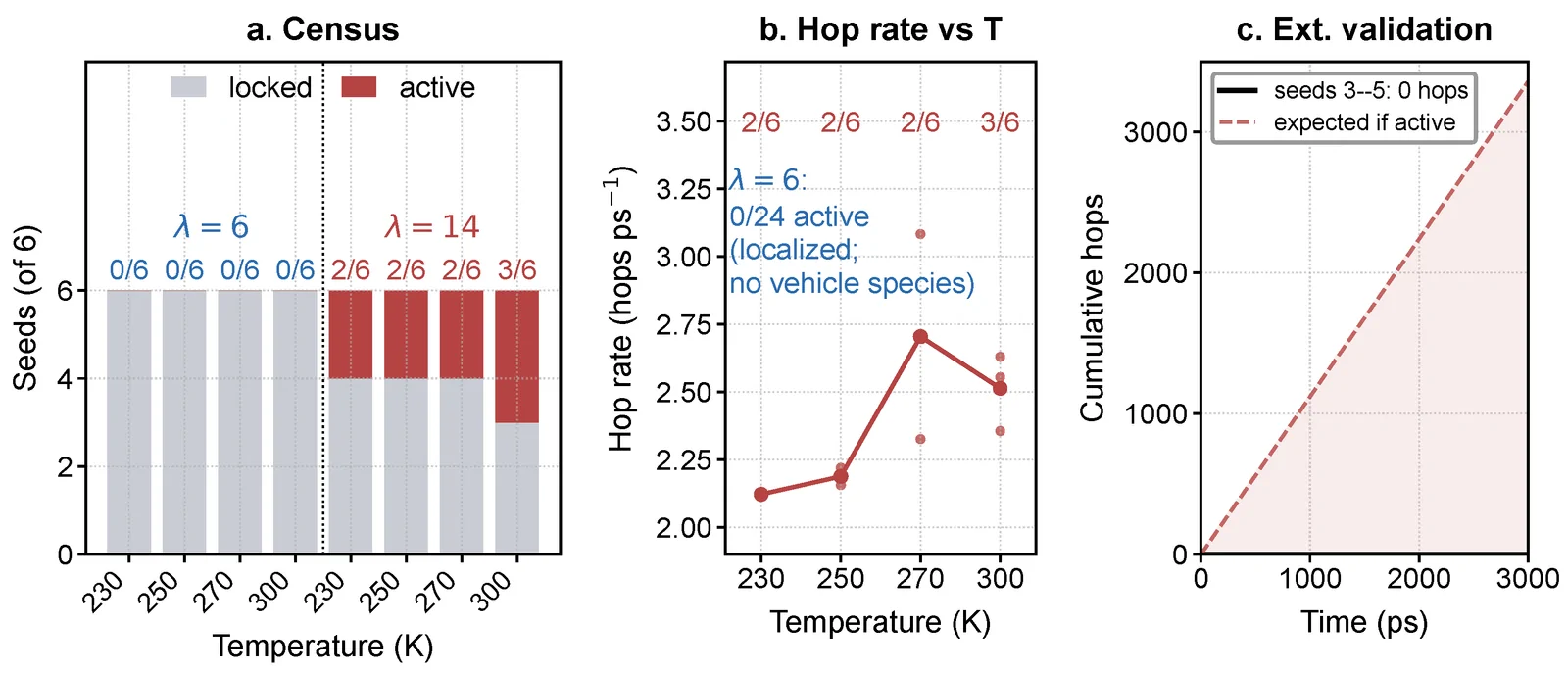
(**a**) Census of Grotthuss-active and locked trajectories across the 48-seed dataset (active = sustained hopping, ≈2–3 hops/ps throughout the 1 ns window; edgeless bars, coloured = active, grey = locked; a marginal λ = 14, 230 K seed with two isolated events is counted with the locked branch); (**b**) hop rates of the nine stably active λ = 14 trajectories versus temperature at the native 50 fs census resolution (2.1–3.1 hops/ps). Dots = individual seeds, line = mean; a tenth marginal trajectory at 230 K, seed 3, with two hops in 1 ns is omitted; (**c**) extended validation at λ = 14, 230 K (analyzed in Section 3.4): cumulative hops expected for active trajectories at the observed rate (1.12 hops/ps at the original 200 fs cadence, red dashed reference and shaded envelope) versus the observed count of zero over three additional 1 ns runs (seeds 3–5, solid black), confirming that the locked state is not a sampling artifact. All 24 λ = 6 trajectories remain locked at every temperature.

At λ = 6, all 24 trajectories (4 temperatures × 6 seeds, ≈24 ns of aggregate sampling) exhibit exactly zero host-oxygen changes (Figure 8a) at the native 50 fs analysis resolution, so no net proton transfer is resolved on timescales up to 1 ns. A transfer completing and reversing within a single 50 fs interval would not be registered; such an event is however by definition a localization of the proton on its parent oxygen and is therefore consistent with, rather than contrary to, the conclusion drawn here. Sampling at 50 fs is in any case fine relative to the transfer event itself: the host-oxygen residence time on active λ = 14 trajectories is 0.7–0.9 ps (Section 3.2), i.e., 14–18 saved frames per transfer, and coarsening the census to 200 fs is shown below to miss 43–48% of resolved events. Because no hydronium-like motif is detected at λ = 6 at any temperature (below), there is in addition no vehicle species whose translational motion could carry the charge; the nonzero tracer $D_{H^{+}}$ of Table 2 therefore reflects localized motion of the acidic proton within its sulfonate first shell together with the segmental displacement of the sulfonate group itself, and should not be read as vehicle transport of a hydrated proton [11]. The hydrogen-bond picture is quantified in Section 3.2 (Figure 7b). The event-level analysis therefore provides the physically meaningful diagnosis at λ = 6. This result is physically intuitive: at λ = 6, water molecules are predominantly confined within first-shell hydration sites around ${\mathrm{SO}}_{3}^{-}$ groups, forming isolated clusters rather than percolated networks. The absence of continuous hydrogen-bond pathways precludes the multi-step relay required for Grotthuss transport. This finding is consistent with the structural picture established by classical MD simulations, in which water molecules form isolated clusters around sulfonic acid groups without establishing the continuous hydrogen-bond pathways required for structural diffusion [15,18].

Using a 2.5 Å nearest-neighbor criterion (which by construction constrains only the first coordination shell of each sulfonate and does not distinguish a covalently bound ${\mathrm{SO}}_{3}$H proton from a tightly hydrogen-bonded one), every sulfonate group retains at least one hydrogen for the entire simulation on all 48 trajectories (mean nearest S–H distance 2.104–2.115 Å; dissociation fraction 0.000). Two qualifications attach to that figure. First, it is a proximity criterion rather than a covalent-bond criterion, so it reports the absence of any sulfonate left without a nearby proton, not the absence of ionization. Second, with only ten exchange sites per cell a count of 0/10 carries a Clopper–Pearson 95% upper bound of ≈26%, so a partially ionized population of order 10–25% cannot be excluded by the present statistics. Reactive simulations of Nafion with 320 exchange sites per cell and a purpose-trained ReaxFF find that protonated sites persist at every hydration level from λ = 0 to 20, their proportion decaying exponentially with λ but never vanishing [31]. The present model, which starts from fully protonated ${\mathrm{SO}}_{3}$H and retains a nearby proton at every site throughout, is consistent with that finding; together the two studies contradict the assumption of complete ionization above a fixed threshold λ that is commonly adopted in classical simulations of these membranes. Two complementary residence-time measures make the mechanistic contrast between the two hydration levels explicit. The host-oxygen residence time of the excess proton (the interval before a Grotthuss host change) is, at λ = 6, the full 1 ns window at every temperature, since no host change ever occurs; correspondingly, no hydronium-like ($H_{3}O^{+}$) coordination motif is detected. At λ = 14, by contrast, the stably active trajectories sustain a mean of 1.1–$1.3\;H_{3}O^{+}$-like defects per frame with sub-picosecond host residence (Figure 7c), the excess protons residing on water molecules within the sulfonate second shell rather than as fully separated ions, while the locked trajectories show neither. Because at λ = 6 the acidic-proton identity is fixed for the whole trajectory, the λ = 6 conductivity and activation energy reported in Section 3.1 are upper-bound quantities associated with localized acidic-proton motion and segmental displacement of the sulfonate group rather than long-range charge transport.

At λ = 14, the picture is markedly more complex, and we distinguish **stably active** trajectories (a sustained hop rate above ≈2 hops/ps throughout the window) from **marginal** and **locked** ones. At 300 K, three of six seeds are stably active (2.36–2.63 hops/ps at the native 50 fs resolution), while the remaining three are locked (zero hops). At 270 K, two seeds are stably active (2.33–3.08 hops/ps); at 250 K, two seeds are stably active (2.16–2.22 hops/ps); and at 230 K, two seeds are stably active (2.12 hops/ps each), with a third, marginal seed (seed 3) exhibiting only two isolated events over 1 ns (0.002 hops/ps) that do not constitute sustained conduction. Counting only sustained hopping, the sustained-active fraction is therefore 3/6 at 300 K and 2/6 at 270 K and below (the marginal 230 K seed 3 grouped with the locked branch for all rate statistics); nine trajectories in total are stably active, and the locked fraction grows as temperature decreases. The seed-resolved hop rates of these nine stably active trajectories are collected as a function of temperature in Figure 8b; the extended-validation panel (Figure 8c), which tests whether the locked branch reflects undersampling rather than genuine kinetic arrest, is analyzed in Section 3.4.

Two qualifications on the hydration dependence follow from these statistics. First, only two hydration levels were simulated, so the data establish two distinct regimes at λ = 6 and λ = 14; the crossover between them lies somewhere in the range 6 < λ ≤ 14 and remains unresolved. Second, the switch is better described in a continuous structural variable than in λ itself. At fixed λ = 14 the hopping competence is already distributed rather than binary—the stably-active fraction is 3/6 at 300 K and 2/6 at 270 K and below, and one 230 K trajectory is marginal—while the hydrogen-bond connectivity varies continuously between the two hydration levels (hydrogen bonds per water ≈1.0 at λ = 6 versus ≈1.4 at λ = 14; running $O_{w}$–$O_{w}$ coordination ≈2.7 versus ≈4.5, Section 3.2). We therefore regard hydrogen-bond connectivity, which the present simulations sample continuously, as the order parameter of the mechanistic switch, with λ acting as a coarse proxy for it. This choice also improves transferability, since a threshold located precisely on the model’s λ scale, whose equivalent weight is ≈1498 g/mol against ≈1100 g/mol for commercial Nafion, would not transfer directly to membranes of lower equivalent weight (Section 2.2). An independent study of Nafion at λ = 3, 9, 15 and 20 likewise places the hydration-limited regime below λ≈ 9, although in the vehicle mechanism and with a non-dissociative hydronium model [34].

The hop rates correspond to mean waiting times of 0.32–0.47 ps between successive host changes at the native 50 fs resolution, consistent with the sub-picosecond Grotthuss hopping timescales characterized in molecular-level studies of aqueous proton transport [52]. These waiting times are shorter than the 0.7–0.9 ps host-oxygen residence times reported in Section 3.2 (Figure 7c) because the two quantities count different events: the residence time is the net interval an oxygen retains the excess proton, whereas the hop rate counts every change of the single argmax host, including the immediate back-hops quantified below (≈31%) and transient switches among the 1.1–1.3 coexisting hydronium-like defects per frame (Section 2.4). The two measures therefore differ by roughly the back-hop factor and should not be equated. If each observed hop contributed independently to diffusion, an uncorrelated random-walk estimate *D* ≈ $\Gamma d^{2}/6$ with Γ ≈ 2.1–3.1 hops/ps and *d* ≈ 2.7–3.0 Å (the split first O–O shell, Figure 5a; see Section 3.2) would yield *D* ≈ (2.6–4.7) × $10^{-8}\;m^{2}$/s, two or more orders of magnitude above the measured tracer diffusion coefficients at λ = 14 (0.9–2.4 × $10^{-10}\;m^{2}$/s). The vast majority of transfer events must therefore reverse almost immediately: the excess proton rattles back and forth within short-lived hydrogen-bonded complexes, and only a small fraction of hops escape the local cage to contribute to net displacement. The 50 fs census provides direct quantitative evidence for this reversibility: across the nine stably active trajectories, 29–34% of hops return to the previous host oxygen within 200 fs (back-hop fraction; mean ≈0.31), and the 200 fs cadence of the originally reported counts misses 43–48% of the events resolved at 50 fs. Because the tracer diffusion coefficients are unaffected by the analysis resolution, the effective anti-correlation between successive hops is even stronger than the uncorrelated random-walk estimate implies; such strongly anti-correlated hopping invalidates a simple uncorrelated random-walk interpretation of proton diffusion [44], echoing the correlated proton hopping dynamics characterized in recent simulations of liquid water [53]. The present event-level statistics thus provide, in a confined PFSA environment, a direct microscopic counterpart to the Grotthuss–vehicle anti-correlation identified in concentrated acid solutions [12]. The same cage-and-escape picture has been reported independently for the vehicle mechanism in Nafion, where hydrated protons alternating between cage rattling and cage jumping conduct far better than protons confined to rattling alone [34], and for back-and-forth proton motion in Grotthuss-type transport by first-principles simulation [35]. The present census supplies the reactive, event-resolved counterpart on the Grotthuss side, quantifying both the reversibility (back-hop fraction 0.29–0.34) and the small per-event charge displacement (0.42 Å) that a non-dissociative hydronium model cannot resolve.

An independent confirmation of this picture comes from the excess-charge-center (CEC) analysis (Section 2.4). Across the nine stably active trajectories, the per-hop CEC displacement is tightly clustered, with per-trajectory medians spanning 0.37–0.46 Å (grand mean 0.42 Å across the nine stably active trajectories), roughly an order of magnitude smaller than the O–O nearest-neighbor spacing (2.7–3.0 Å): a typical registered hop relocates the tracer identity while leaving the charge center essentially stationary, the defining signature of Grotthuss-type shuttling in which the excess charge is shared across a hydrogen-bonded complex rather than translated. The hop counts recovered by the CEC analysis are identical to those of the 50 fs census on all nine stably active trajectories, confirming that both analyses rest on the same event definition. The CEC mean-square displacement yields charge-diffusion coefficients $D_{\mathrm{CEC}}$ = (4.3 × $10^{-10}$–7.8 × $10^{-9}$) $m^{2}$/s on the active trajectories, about one order of magnitude above the tracer $D_{H^{+}}$ at the same conditions. These values are evaluated over the short Fickian window (30–50 ps), the same window used for the bulk-water benchmark of Table 3, because the charge-center mean-square displacement saturates at longer lags in confined trajectories: over 100–500 ps windows the CEC MSD is non-diffusive, with fitted slopes near zero or negative in several active trajectories, reflecting cage confinement of the charge defect rather than long-range charge migration. $D_{\mathrm{CEC}}$ is therefore an effective short-time charge diffusivity, not a converged long-time value, and should not be extrapolated to the macroscopic conductivity without the window qualification. The reported $H_{R}=D_{\mathrm{CEC}}/D_{H^{+}}$ ≈ 3–55 is evaluated against the condition-mean $D_{H^{+}}$; individual-seed ratios reach ≈2–240, but these extreme values arise from the vanishingly small tracer diffusivities at 230–250 K (Section 3.1) and are quoted only as a range bound rather than as physical Haven ratios. Net charge transport is therefore dominated by the small fraction of hops that escape the local cage, consistent with the back-hop statistics. Conversely, on the locked λ = 14 trajectories and on all λ = 6 trajectories, $D_{\mathrm{CEC}}$ remains in the ∼$10^{-11}$–$10^{-9}\;m^{2}$/s range, comparable to the tracer values, so that in the absence of hopping the Nernst–Einstein relation with tracer diffusion is internally consistent. The correlation error quantified by $H_{R}$ implies that the tracer-based conductivities of Table 2 should be read as order-of-magnitude references for the stably active subset (Haven-ratio-type corrections of up to ∼10×), and as upper bounds at λ = 6, where no hops occur and no hydronium-like vehicle exists, so that the tracer motion is localized rather than long-range charge transport [42,43]. This distinction follows the MS-EVB treatment of aqueous proton transport, in which the charge centroid rather than any individual hydrogen defines the diffusing species [40,41].

Despite the presence of a percolated water network at λ = 14, a fraction of the trajectories at every temperature, including 230 K, fails to exhibit any hopping whatsoever, and this locked fraction grows as temperature decreases (three of six seeds locked at 300 K, four of six at 270 K and below). The coexistence of locked and stably active trajectories at identical thermodynamic conditions is trajectory- and configuration-dependent: the hydrogen-bond network can reside either in a rigid, hopping-incompetent state or in a dynamically reorganized, hopping-competent state. Whether this bimodality reflects a genuinely metastable landscape or, alternatively, the persistence of distinct initial configurations over the nanosecond observation window was tested directly by same-configuration velocity-reseed controls. From one active and one locked λ = 14, 300 K configuration, three independent velocity reseedings were each propagated for 1 ns at the identical thermodynamic state: all three reseeds of the active configuration reactivated immediately (1.89–2.18 hops/ps; back-hop fraction 0.29–0.30), while all three reseeds of the locked configuration remained fully locked (zero hops; binomial 95% Clopper–Pearson intervals of [0.29, 1] for 3/3 activation and [0, 0.71] for 0/3 locking). All six reseeded trajectories therefore reproduced the fate of their parent configuration (6/6; 95% CI [0.54, 1]), establishing that on the nanosecond timescale the stably active/locked identity is configuration-determined rather than set by the instantaneous velocities. The bimodality accordingly reflects the persistence of distinct prepared hydrogen-bond-network configurations; whether these configurations are separated by barriers that would permit spontaneous interconversion on substantially longer (tens-of-nanoseconds) timescales remains open. The activation of Grotthuss transport requires not merely the existence of water channels but also sufficient thermal energy for water reorientation between successive proton transfers. At 230 K this reorientation step becomes a severe kinetic bottleneck: configurations whose hydrogen-bond network lacks the thermal flexibility to reorganize remain locked for the entire nanosecond window, whereas configurations that establish a favorable relay geometry continue to conduct even at 230 K. This picture is consistent with molecular-level studies of supercooled water, in which dynamically arrested, low-mobility regions grow on cooling and govern relaxation [54]. Under nanoscale confinement, water relaxation retains an Arrhenius temperature dependence with evidence of a fragile-to-strong crossover near 228 K [55], and the glass transition of confined water sets in over the 170–200 K range rather than at the bulk value [56]; ice nucleation from room-temperature water has itself been predicted directly in machine-learning simulations [57]. The locked configurations observed here can therefore be viewed as rigidified domains of the hydrogen-bond network in which the reorientation step prerequisite to proton relay is dynamically suppressed. This mechanistic partition is consistent with the transport statistics of Section 3.1. The sub-diffusive majority (38 of the 48 trajectories, responsible for the sub-linear MSD plateaus of Figure 1) spans both mechanistic branches: most λ = 6 trajectories, in which the tracer protons undergo localized motion on their sulfonate groups, nearly all locked λ = 14 trajectories, and most of the stably active ones, whose atom-indexed tracer protons do not follow the migrating charge (Section 2.4); the ten short Fickian windows identified in Section 3.1 (2–35 ps) are distributed across both hydration levels. Net charge transport is therefore controlled by sustained relay (the $D_{\mathrm{CEC}}$ contrast quantified above), which in turn requires a hydrogen-bond network flexible enough to reorganize between successive transfers.

**Figure 9:**
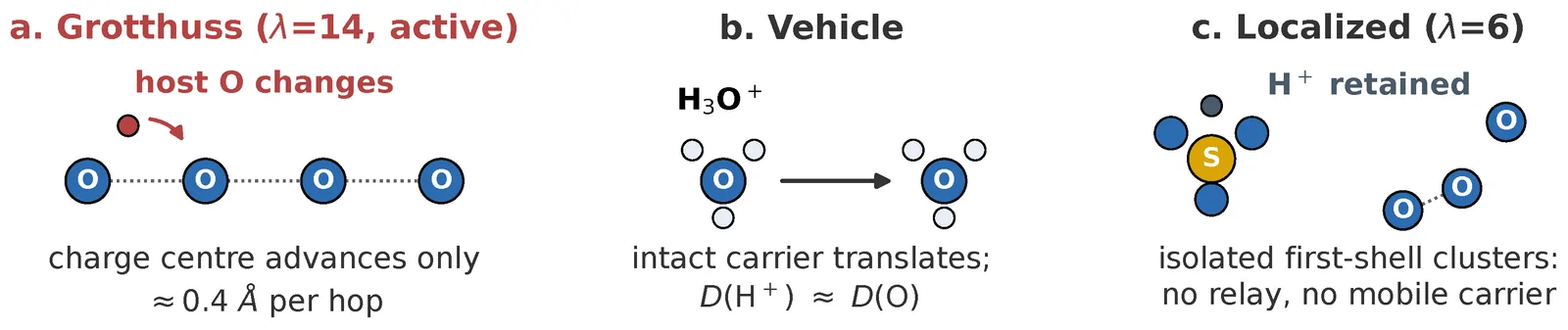
Schematic of the three proton-transport regimes distinguished by the present analysis. (**a**) Grotthuss shuttling (stably active λ = 14 trajectories): the excess proton relocates between host oxygens along the hydrogen-bond network; the charge centre advances only ≈0.4 Å per hop, so net transport requires the minority of hops that escape the local cage. (**b**) Vehicle transport: an intact $H_{3}O^{+}$ translates together with its solvation shell, so the tracer and the carrier move together and $D_{H^{+}}\approx D_{O}$. (**c**) Localized regime (λ = 6): the acidic proton is retained on its sulfonate group and water forms isolated first-shell clusters, so neither a Grotthuss relay nor a mobile vehicle exists; the nonzero tracer diffusivity reflects localized proton motion and segmental displacement of the sulfonate group.

The reactive simulation approach adopted here provides a methodological advantage over prior classical MD studies. Because reactive ReaxFF dynamics allow explicit bond-breaking and bond-forming events [58], individual Grotthuss hops can be counted directly rather than inferred from differences between $D_{H^{+}}$ and $D_{O}$, an indirect proxy that, as Figure 2b shows, becomes unreliable when the two diffusion coefficients are comparable. The event counts reported in Figure 8a therefore provide a direct, unambiguous quantification of the mechanism. This capability parallels the reactive simulations of Mabuchi and Tokumasu [25], who demonstrated that a reactive MD model can capture the hydration-dependent proton transport behavior that fixed-topology force fields cannot represent, and extends prior ab initio analyses of proton hopping in model proton-exchange membranes [29] to larger spatial and temporal scales. Figure 9 summarizes the three regimes that the present analysis distinguishes: Grotthuss shuttling, in which the host oxygen changes while the charge centre advances only ≈0.4 Å per hop; vehicular transport, in which an intact hydronium translates with its solvation shell so that $D_{H^{+}}\approx D_{O}$; and the localized regime identified at λ = 6, in which the acidic proton is retained on its sulfonate group and water occupies isolated first-shell clusters, so that neither a relay nor a mobile carrier exists.

**Force-field anchor: excess-proton diffusion in bulk water:** To benchmark the proton-transfer kinetics of ReaxFF_PEMFC in a system with known transport physics, a periodic cell of 256 $H_{2}$O molecules plus one excess proton ($H_{3}O^{+}$) was simulated at 300 K in four independent 100 ps seeds, and the same hop census, CEC analysis, and MSD protocol as in the membrane systems were applied (Table 3). Per-seed CEC diffusivities span (1.3–6.3) × $10^{-9}\;m^{2}$/s; one seed exhibits an anomalously low value (1.3 × $10^{-9}\;m^{2}$/s, log–log MSD slope 0.29) despite a normal hop count (2.17 hops/ps), a rare trajectory in which the CEC remains confined to a sub-region of the box, and the median is therefore used as the central estimate, $D_{\mathrm{CEC}}$ = 5.3 × $10^{-9}\;m^{2}$/s, within a factor of ≈1.7 of the experimental excess-proton diffusivity of ≈9 × $10^{-9}\;m^{2}$/s in bulk water [7,44]; the three well-behaved seeds alone give 5.6 × $10^{-9}\;m^{2}$/s. The oxygen (vehicle) diffusion coefficient $D_{O}$ = 0.75 × $10^{-9}\;m^{2}$/s (median; per-seed 0.66–0.87) likewise reproduces the factor-of-two-to-three underestimation of water self-diffusion typical of ReaxFF water models relative to the experimental ≈2.3 × $10^{-9}\;m^{2}$/s. The Grotthuss enhancement $D_{\mathrm{CEC}}$/$D_{O}$ ≈ 7 reproduces the dominance of structural (Grotthuss) diffusion over vehicle transport in bulk water. At the same 1.30 Å host criterion, the hop census returns 1.86–2.17 hops/ps with 20–25% back-hops and a median per-hop CEC displacement of ≈2.6 Å (three seeds; seed 2 excluded as a periodic-wrap outlier), closely comparable to the 2.1–3.1 hops/ps measured in the active λ = 14 membrane trajectories; the hydronium host remains three-coordinated (*n* = 3) in 88–92% of frames, as expected for $H_{3}O^{+}$ in bulk water. The proton-transfer barrier implicit in ReaxFF_PEMFC therefore yields diffusion coefficients and elementary hop rates of the correct order of magnitude in a well-characterized system, providing an external anchor for the event-level statistics of Section 3.1, Section 3.2 and Section 3.3. That barrier is nevertheless implicit in the force-field parametrization and is validated here only against this bulk-water benchmark, so the absolute hop rates (2.1–3.1 hops/ps) and the frequency of the active/locked split (3/6 at 300 K) are force-field-specific quantities rather than transferable material constants.

### 3.4. Low-Temperature Grotthuss Arrest: Extended Validation and Density Sensitivity

To rule out the possibility that the zero-hop trajectories at 230 K reflect undersampling rather than genuine kinetic arrest, extended simulations were performed (Figure 8c): three trajectories (seeds 3, 4, 5) at λ = 14 and 230 K were continued for an additional 1 ns each (total 2 ns per trajectory; the extension segments were stored and analyzed at the native 50 fs resolution). Across 3000 ps of combined additional sampling, no hopping events were observed in any of the three extended trajectories. The per-window hop count (0–250 ps, 250–500 ps, 500–750 ps, 750–1000 ps) remained identically zero. This confirms that the locked state persists on the extended timescale: within these configurations, the hydrogen-bond network is rigidified at 230 K such that water reorientation, the prerequisite for Grotthuss relay, is kinetically inaccessible on nanosecond timescales, even though stably active trajectories at the same temperature (seeds 1, 2) continue to hop. The three additional nanoseconds of sampling with zero events thus strongly constrain the possibility that the locked state reflects insufficient observation time. The same test was applied at 300 K, where thermal activation is most favorable: two locked λ = 14 trajectories (seeds 4 and 5) were each continued for a further 2 ns (3 ns per trajectory in total) and re-analyzed at the full 50 fs resolution, again with zero hopping events in either trajectory. In total, the locked branch is now supported by ≈7 ns of zero-event sampling at 300 K (the three original 1 ns locked seeds plus the two 2 ns extensions of seeds 4 and 5) and ≈6 ns at 230 K (the three original 1 ns locked seeds, 4–6, plus the three 1 ns extensions of seeds 3–5), where seeds 4 and 5 are locked over both the original and extended windows and the marginal seed 3 (two isolated events in its first nanosecond) recorded zero further events throughout its 1 ns extension; every extension segment is therefore itself event-free. Per trajectory, the 2 ns extensions bound any residual hop rate below 5 × $10^{-4}$ hops/ps (a 1/*T* upper bound), about four orders of magnitude below the active-trajectory rates. Within the tested window, slow activation of locked configurations is therefore not observed, although activation on timescales beyond a few nanoseconds cannot be excluded.

No ice-like ordering is observed or claimed at any temperature studied, and the term “arrest” used in this section refers exclusively to the kinetics of the proton relay, not to a phase transition of the water. All water in the model is confined or bound hydration water: at λ = 6 the 60 water molecules occupy first-shell hydration sites around the ten sulfonate groups, with ≈1.0 hydrogen bonds per water and a running $O_{w}$–$O_{w}$ coordination of only ≈2.7 to the first minimum, while at λ = 14 the coordination reaches ≈4.5 but with a split, confinement-broadened first shell and only ≈1.4 hydrogen bonds per water. Neither condition corresponds to the fourfold tetrahedrally ordered network with long-range correlation that characterizes ice Ih, and no bulk-like free-water population exists at either hydration level in which a crystalline nucleus could form. This is physically appropriate to the regime addressed: during cold start, ice forms in the catalyst layer and at interfaces [48,49], neither of which is represented in a membrane-interior model. The temperature range studied is likewise consistent with confined water remaining mobile below the bulk melting point, since the glass transition of confined water sets in over 170–200 K [56] and 230 K lies above it, in the supercooled-but-mobile regime [55]. Finally, the results are internally inconsistent with a frozen phase: two of six λ = 14 configurations sustain 2.12 hops/ps at 230 K over the full 1 ns window, which a crystalline ice phase could not support. The coexistence of sustained hopping and complete kinetic arrest at identical temperature and hydration is a dynamical heterogeneity characteristic of a supercooled liquid rather than of a uniformly ordered solid; the supercooled-water studies cited in this section [54,55,56] are invoked strictly as a dynamical analogy and not as evidence of a phase transition.

The fixed-cell protocol (Section 2.3) held the cell at its as-built dimensions, giving densities (≈1.0–1.1 ${g/\mathrm{cm}}^{3}$) below those of hydrated PFSA membranes at comparable hydration [59]. The under-dense cell affects the reported coefficients in three ways: the excess free volume raises the computed diffusion coefficients relative to a fully densified membrane; the carrier number density *n* = 10/$V_{\mathrm{cell}}$ is correspondingly reduced, so that the two effects partially compensate in the Nernst–Einstein conductivity (Section 3.1); and the enlarged water–water separation lowers the hydronium coordination numbers of Section 3.2. To test whether this density deficit affects the mechanism rather than merely its rates, the λ = 14 system was re-equilibrated under NPT (Berendsen barostat, 1 bar, 500 ps) at 300 and 230 K, expanding to ρ ≈ 0.64 and 0.81 ${g/\mathrm{cm}}^{3}$. At 300 K the NPT hop rate (2.35 hops/ps) remained within ≈11% of the fixed-cell value (2.63 hops/ps) despite a ≈41% density reduction, whereas at 230 K no hops were resolved within the 500 ps NPT window (versus 2.1 hops/ps over 1 ns in the fixed cell) at a smaller density reduction of ≈25%. First, the barostat moved the density away from the experiment: the fixed cell is already under-dense at ≈1.08 ${g/\mathrm{cm}}^{3}$ and NPT expanded it further to 0.64 and 0.81 ${g/\mathrm{cm}}^{3}$, whereas dry PFSA is ≈2.05 ${g/\mathrm{cm}}^{3}$ and a liquid-equilibrated membrane approaches ≈1.5 ${g/\mathrm{cm}}^{3}$ [59]. This test therefore cannot bound the effect of the density deficit; it can only show that the hopping mechanism is robust to rarefaction. Second, the outcome does not track the magnitude of the density change: the 300 K run expanded more yet lost only ≈11% of its hop rate, while the 230 K run expanded less yet lost all resolved activity. Density alone therefore does not determine the active/locked outcome. Third, the 230 K null result is inconclusive rather than confirmatory, since it rests on a 500 ps window against 1 ns in the fixed cell, on a single starting configuration at a temperature where three of six seeds are already locked and a fourth is marginal, and on a Berendsen barostat that does not generate a correct ensemble. The λ = 14 cell was also compressed to ρ = 1.75 ${g/\mathrm{cm}}^{3}$ and propagated for 1 ns in NVT at 300 K and at 230 K, so that the as-built (≈1.08 ${g/\mathrm{cm}}^{3}$) and compressed cells bracket the liquid-equilibrated ≈1.5 ${g/\mathrm{cm}}^{3}$ from below and above. Transfers counted with the covalent criterion of Section 2.4 give 1.39 hops/ps at 230 K, with a hydronium-like defect in 99.9% of frames, and 0.044 hops/ps at 300 K, with a hydronium-like defect in 2.9% of frames. Every event at both temperatures has an O–O separation of 2.2–2.8 Å and none exceeds 3.5 Å. These two runs are single configurations of the same model, one sustaining transfer and the other largely not, in the same way as the active and locked trajectories of Section 3.3. At 1.75 ${g/\mathrm{cm}}^{3}$ a hop remains a short-range transfer through a hydronium-like defect, as at the as-built density. The mechanistic conclusions (absence of Grotthuss activity at λ = 6 and the bimodal active/locked landscape at λ = 14, both governed by water connectivity and thermal fluctuations) therefore remain unchanged, whereas the 230 K quantitative rates carry the largest density-related uncertainty. Independent of these model-specific quantities, four conclusions are expected to hold for PFSA membranes generally: the absence of resolved Grotthuss transfer once water forms isolated first-shell clusters at low hydration (≈1.0 hydrogen bonds per water); the hydration-controlled rather than temperature-controlled character of the mechanistic switch (λ = 6 remains hop-free even at 300 K); the reversal of most transfer events, so that net transport is carried by a minority of cage-escaping hops; and the failure of the diffusivity-contrast proxy in this regime, since $D_{O}\approx D_{H^{+}}$ throughout.

## 4. Conclusions

ReaxFF reactive molecular dynamics resolved how hydration level and temperature govern the proton-transport mechanism in a PFSA membrane, across λ = 6 and λ = 14, four temperatures (230–300 K), and 48 independent trajectories of ≈1 ns each. Counting proton-transfer events directly, rather than inferring the mechanism from tracer–oxygen diffusion contrasts, reveals two distinct mechanistic regimes at λ = 6 and λ = 14, with the crossover between them lying somewhere in the range 6 < λ ≤ 14. No intermediate hydration was simulated, so the position of the crossover remains unresolved, and that range is quoted on a model scale whose equivalent weight (≈1498 g/mol) exceeds that of commercial Nafion (≈1100 g/mol).

The main findings are as follows. At λ = 6, no Grotthuss hopping is resolved at any temperature, the acidic protons remain localized on their sulfonate groups, and no hydronium-like motif is detected; there is consequently no vehicle species to carry the charge, and the nonzero tracer diffusivity reflects localized acidic-proton motion together with segmental displacement of the sulfonate group rather than vehicle transport. The corresponding diffusion-based activation energy (0.09 eV) is an order-of-magnitude upper bound. At λ = 14, nine of the twenty-four trajectories are stably active across the four temperatures (2.1–3.1 hops/ps) while the remainder stay kinetically locked over the observation window. Same-configuration velocity-reseed controls establish that this stably active/locked identity is configuration-determined on the nanosecond timescale, and charge-center analysis attributes net transport to the small fraction of cage-escaping hops. Hydronium coordination peaks at CN = 1, an under-coordination consistent with first-shell confinement. No ice-like ordering is observed or claimed at any temperature; the arrested configurations are rigidified hydrogen-bond networks in a supercooled-but-mobile regime, not a frozen phase.

For PEMFC cold-start operation, membrane designs preserving local hydration at the catalyst–membrane interface should sustain structural diffusion, and the apparent λ = 6 activation energy serves as an order-of-magnitude reference for continuum cold-start models. At λ = 14, shifting the stably active/locked balance toward the conducting branch through thermal or morphological control could mitigate the conductivity loss associated with the locked fraction. At λ = 6, by contrast, no temperature in the range studied activates hopping, so thermal management alone cannot restore structural diffusion and rehydration is required—a distinction of direct consequence for cold-start strategy, in which membrane temperature and hydration change simultaneously. Each quantitative value carries the qualification stated where it is reported. The absolute coefficients are order-of-magnitude values for an under-dense, high-equivalent-weight fixed cell (Section 2.2 and Section 3.4), and the proton-transfer barrier is validated only against the bulk-water benchmark (Section 3.3, Table 3). The event statistics are single-centre, per-defect quantities obtained over a nanosecond window that cannot exclude rare locked–stably active interconversion (Section 2.4 and Section 3.3). These bound the quantitative rates, most notably at 230 K, while leaving the mechanistic picture intact. Diagnosing the mechanism from an explicit hop census rather than from the $D_{H^{+}}$–$D_{O}$ contrast is the transferable element of this work, and we expect it to apply wherever proton transport in a confined, low-hydration environment must be characterized.

## Figures and Tables

**Table 1:** Parameters of the PFSA membrane models.

Model	Total Atoms	$H_{2}$O	${\mathrm{SO}}_{3}$H	λ	$n_{\mathrm{carrier}}$ ($H^{+}$)
λ = 6	1090	60	10	6	10
λ = 14	1330	140	10	14	10

**Table 2:** Proton transport properties (mean ± s.d. over six seeds per condition). $D_{H^{+}}$ is the tracer (self-)diffusion coefficient of the ten sulfonic-acid protons from the linear MSD regime; $\sigma_{H^{+}}$ is derived from the Nernst–Einstein relation (Equation (6)) with *n* = 10 carriers per cell. Arrhenius fits over 230–300 K: $E_{a}\left(\sigma \right)$ = 0.071 eV ($R^{2}$ = 0.670) and 0.062 eV ($R^{2}$ = 0.855) for λ = 6 and 14; $E_{a}\left(D_{H^{+}}\right)$ = 0.094 eV ($R^{2}$ = 0.776) and 0.085 eV ($R^{2}$ = 0.911). The tabulated $\sigma_{H^{+}}$ are tracer-based, order-of-magnitude values: on the stably active λ = 14 subset the excess-charge-center analysis (Section 3.3, which also defines the stably active, marginal, and locked trajectory classes) implies Haven-ratio-type corrections of up to ≈10×, whereas at λ = 6, where no hopping events occur, they are apparent upper bounds set by localized acidic-proton motion. Only 10 of the 48 trajectories reach a Fickian regime within 1 ns, so both columns are effective values rather than converged material constants: $D_{H^{+}}$ carries a per-trajectory window sensitivity of factors 3–13 (Section 3.1), averaged over in the six-seed statistics, and $\sigma_{H^{+}}$ is a lower bound wherever Grotthuss hopping operates (Section 2.4 and Section 3.1).

λ	*T* (K)	$D_{H^{+}}$ ($10^{-11}\;m^{2}\;s^{-1}$)	$\sigma_{H^{+}}$ (S ${\mathrm{cm}}^{-1}$)	Stably Active Seeds (of 6)
6	230	7.632 ± 5.898	0.0023 ± 0.0018	0
6	250	6.417 ± 2.805	0.0018 ± 0.0008	0
6	270	14.729 ± 8.433	0.0038 ± 0.0022	0
6	300	19.688 ± 11.920	0.0045 ± 0.0027	0
14	230	8.559 ± 7.038	0.0026 ± 0.0021	2
14	250	10.310 ± 7.476	0.0028 ± 0.0021	2
14	270	12.485 ± 9.054	0.0032 ± 0.0023	2
14	300	23.749 ± 9.824	0.0055 ± 0.0023	3

**Table 3:** Pure-water benchmark of excess-proton transport (256 $H_{2}$O + one excess proton, 300 K, four independent 100 ps seeds). $D_{\mathrm{CEC}}$: excess-charge-center diffusion coefficient from the ensemble MSD (Fickian window 30–50 ps); $D_{O}$: oxygen (vehicle) diffusion coefficient; hop rate and back-hop fraction at the 1.30 Å host criterion, 50 fs resolution. The two outliers are distinct and affect different metrics: seed 4 is excluded only from the $D_{\mathrm{CEC}}$ central estimate (confined-CEC trajectory, normal hop count but sub-diffusive CEC motion, log–log slope 0.29), whereas seed 2 is excluded only from the median per-hop CEC displacement as a periodic-wrap artefact (Section 3.3); all four seeds are retained for the hop-rate and back-hop statistics. The median is used as the central estimate. Experimental references at ≈298–300 K: $D_{H^{+}}$ ≈ 9 × $10^{-9}\;m^{2}$/s [7,44]; $D_{\mathrm{water}}$ ≈ 2.3 × $10^{-9}\;m^{2}$/s.

Seed	$D_{\mathrm{CEC}}$ ($10^{-9}\;m^{2}\;s^{-1}$)	$D_{O}$ ($10^{-9}\;m^{2}\;s^{-1}$)	Hop Rate (${\mathrm{ps}}^{-1}$)	Back-Hop (%)
1	6.3	0.74	1.86	20.4
2	5.1	0.87	1.86	23.7
3	5.5	0.66	2.17	25.3
4 ^*a*^	1.3	0.76	2.17	22.6
Median	5.3	0.75	2.0 ± 0.2	23.0

^*a*^ Rare confined-CEC trajectory (see caption).

## Data Availability

The data supporting the findings of this study are available from the corresponding author on reasonable request.

## References

[1] Jiao K., Xuan J., Du Q., Bao Z., Xie B., Wang B., Zhao Y., Fan L., Wang H., Hou Z. (2021). Designing the next generation of proton-exchange membrane fuel cells. Nature.

[2] Yang X., Sun J., Meng X., Sun S., Shao Z. (2023). Cold start degradation of proton exchange membrane fuel cell: Dynamic and mechanism. Chem. Eng. J..

[3] Huang D., Zhang Y.-D., Shan S.-Y. (2026). A review on supercooled water-ice phase transition within the cathode catalyst layer of proton exchange membrane fuel cells during cold-start. Electrochim. Acta.

[4] Liu P., Xu S. (2023). A review of low-temperature proton exchange membrane fuel cell degradation caused by repeated freezing start. Int. J. Hydrogen Energy.

[5] Jia J., Liu K., Zuo T., Song D., Wang N., Hu S., Wei X., Che Q. (2022). Enhancing proton conductivity at subzero temperature through constructing the well-ordered structure based on carbon dots. J. Membr. Sci..

[6] Zhao J., Song D., Jia J., Wang N., Liu K., Zuo T., Che Q. (2022). Constructing proton exchange membranes with high and stable proton conductivity at subzero temperature through vacuum assisted flocculation technique. Appl. Surf. Sci..

[7] Agmon N. (1995). The Grotthuss mechanism. Chem. Phys. Lett..

[8] Tuckerman M., Laasonen K., Sprik M., Parrinello M. (1995). Ab initio molecular dynamics simulation of the solvation and transport of hydronium and hydroxyl ions in water. J. Chem. Phys..

[9] Schröder M., Gatti F., Lauvergnat D., Meyer H.-D., Vendrell O. (2022). The coupling of the hydrated proton to its first solvation shell. Nat. Commun..

[10] Oh K.-I., Kwac K., Kwak K., Cho M. (2026). Concentration-dependent restructuring of the Eigen-Zundel ensemble revealed by a solvation-state map of the hydrated proton. Cell Rep. Phys. Sci..

[11] Kreuer K.D., Paddison S.J., Spohr E., Schuster M. (2004). Transport in proton conductors for fuel-cell applications: Simulations, elementary reactions, and phenomenology. Chem. Rev..

[12] Mabuchi T. (2022). Revealing the anticorrelation behavior mechanism between the Grotthuss and vehicular diffusions for proton transport in concentrated acid solutions. J. Phys. Chem. B.

[13] Ludueña G.A., Kühne T.D., Sebastiani D. (2011). Mixed Grotthuss and vehicle transport mechanism in proton conducting polymers from ab initio molecular dynamics simulations. Chem. Mater..

[14] Vilčiauskas L., Kreuer K.-D. (2011). Comment on “Mixed Grotthuss and vehicle transport mechanism in proton conducting polymers from ab initio molecular dynamics simulations”. Chem. Mater..

[15] Li Y., Li L., Zhang K., Yang Y., Tan J. (2026). Molecular dynamics insights into the influence of temperature and water content on proton exchange membrane transportation for proton exchange membrane fuel cells. Results Eng..

[16] Zhao Y., Wang G., Chen W. (2023). Molecular Dynamic Simulations of Proton and Water Transport Mechanism in a Nafion Pore. Energy Technol..

[17] Wang J., Chen J., Xu Z., Yang X., Ramakrishna S., Liu Y. (2022). Mesoscale hydrated morphology of perfluorosulfonic acid membranes. J. Appl. Polym. Sci..

[18] Bazaid M., Huang Y., Goddard W.A., Jang S.S. (2023). Proton transport through interfaces in nanophase-separation of hydrated aquivion membrane: Molecular dynamics simulation approach. Colloids Surf. A.

[19] Rahbari A., Hartkamp R., Moultos O.A., Bos A., van den Broeke L.J.P., Ramdin M., Dubbeldam D., Lyulin A.V., Vlugt T.J.H. (2022). Electro-osmotic drag and thermodynamic properties of water in hydrated Nafion membranes from molecular dynamics. J. Phys. Chem. C.

[20] Han Z.-Y., Pei S.-P., Yu C.-Y., Zhou Y.-F. (2024). Molecular Dynamics Simulation Studies on the Micromorphology and Proton Transport of Nafion/Ti_3_C_2_T_*x*_ Composite Membrane. Chin. J. Polym. Sci..

[21] Cui R., Li S., Yu C., Zhou Y. (2023). The evolution of hydrogen bond network in Nafion via molecular dynamics simulation. Macromolecules.

[22] van Duin A.C.T., Dasgupta S., Lorant F., Goddard W.A. (2001). ReaxFF: A reactive force field for hydrocarbons. J. Phys. Chem. A.

[23] van Duin A.C.T., Zou C., Joshi K., Bryantsev V., Goddard W.A., Asthagiri A., Janik M.J. (2014). A ReaxFF reactive force-field for proton transfer reactions in bulk water and its applications to heterogeneous catalysis. Computational Catalysis.

[24] Konermann L., Kim S. (2022). Grotthuss Molecular Dynamics Simulations for Modeling Proton Hopping in Electrosprayed Water Droplets. J. Chem. Theory Comput..

[25] Mabuchi T., Tokumasu T. (2018). Relationship between proton transport and morphology of perfluorosulfonic acid membranes: A reactive molecular dynamics approach. J. Phys. Chem. B.

[26] Petersen M.K., Voth G.A. (2006). Characterization of the solvation and transport of the hydrated proton in the perfluorosulfonic acid membrane Nafion. J. Phys. Chem. B.

[27] Mouhat F., Peria M., Morresi T., Vuilleumier R., Saitta A.M., Casula M. (2023). Thermal dependence of the hydrated proton and optimal proton transfer in the protonated water hexamer. Nat. Commun..

[28] Gong S., Shi R., Wang X., Xu X., Gao Q., Zhao S. (2026). Molecular dynamics study on proton transport in Nafion, sulfonated polyethylene, and sulfonated poly(ether ether ketone) membranes. Chin. J. Chem. Eng..

[29] Zelovich T., Winey K.I., Tuckerman M.E. (2021). Hydronium ion diffusion in model proton exchange membranes at low hydration: Insights from ab initio molecular dynamics. J. Mater. Chem. A.

[30] Guo Y., Huang S.-F., Mabuchi T., Tokumasu T. (2023). Analysis of structural and water diffusional properties of ionomer thin film by coarse-grained molecular dynamics simulation. J. Mol. Liq..

[31] Donnelly D.J., Yang M.Y., Dimakis N., Jang S.S., Goddard W.A., Smotkin E.S. (2025). Atomistic characterization of hydration-dependent fuel cell ionomer nanostructure: Validation by vibrational spectroscopy. J. Mater. Chem. A.

[32] Sengupta S., Pant R., Venkatnathan A., Lyulin A.V. (2022). Molecular-Dynamics Modeling of Nafion Membranes. Macromol. Symp..

[33] Qi W., Li Y., Ruan S., Gui X., Xu Y., Lu H. (2026). Atomistic insights into the degradation of perfluorosulfonic acid membranes: A reactive force field molecular dynamics study. PLoS ONE.

[34] Hu F., Li J., Wu Y., Zhang J., Gao M., Zhang J., Zhao X., Gao L., Wu C. (2026). Cage effect-induced intermittent dynamics govern proton conductivity in Nafion proton exchange membrane. ACS Appl. Mater. Interfaces.

[35] Choe Y.-K., Tsuchida E., Ikeshoji T., Yamakawa S., Hyodo S.-a. (2009). Nature of proton dynamics in a polymer electrolyte membrane, Nafion: A first-principles molecular dynamics study. Phys. Chem. Chem. Phys..

[36] Taborosi A., Aoki K., Zettsu N., Koyama M., Nagao Y. (2025). Molecular dynamics simulation of polymer electrolyte membrane for understanding structure and proton conductivity at various hydration levels using neural network potential. Macromolecules.

[37] Wang N., Qu Z., Zhang G. (2022). Modeling analysis of polymer electrolyte membrane fuel cell with regard to oxygen and charge transport under operating conditions and hydrophobic porous electrode designs. eTransportation.

[38] Jiang F., Dafalla A.M., Wei L., Hu F. (2025). Modeling the cold start process of proton exchange membrane fuel cells. Modeling and Numerical Simulation of Proton Exchange Membrane Fuel Cells.

[39] Rappe A.K., Goddard W.A. (1991). Charge equilibration for molecular dynamics simulations. J. Phys. Chem..

[40] Wu Y., Chen H., Wang F., Paesani F., Voth G.A. (2008). An improved multistate empirical valence bond model for aqueous proton solvation and transport. J. Phys. Chem. B.

[41] Marx D., Tuckerman M.E., Hutter J., Parrinello M. (1999). The nature of the hydrated excess proton in water. Nature.

[42] Seshadri A., Hess L.T.M., Yue S. (2026). Exploring the deviation from Nernst–Einstein conductivity in ionic liquids using machine learning. Digit. Discov..

[43] Popov I., Zhu Z., Young-Gonzales A.R., Sacci R.L., Mamontov E., Gainaru C., Paddison S.J., Sokolov A.P. (2023). Search for a Grotthuss mechanism through the observation of proton transfer. Commun. Chem..

[44] Arntsen C., Chen C., Calio P.B., Li C., Voth G.A. (2021). The hopping mechanism of the hydrated excess proton and its contribution to proton diffusion in water. J. Chem. Phys..

[45] Cappadonia M., Erning J.W., Niaki S.M.S., Stimming U. (1995). Conductance of Nafion 117 membranes as a function of temperature and water content. Solid State Ion..

[46] Sone Y., Ekdunge P., Simonsson D. (1996). Proton conductivity of Nafion 117 as measured by a four-electrode AC impedance method. J. Electrochem. Soc..

[47] Zawodzinski T.A., Derouin C., Radzinski S., Sherman R.J., Smith V.T., Springer T.E., Gottesfeld S. (1993). Water uptake by and transport through Nafion^®^ 117 membranes. J. Electrochem. Soc..

[48] Liang J., Fan L., Du Q., Yin Y., Jiao K. (2023). Ice Formation during PEM Fuel Cell Cold Start: Acceptable or Not?. Adv. Sci..

[49] Schmitz M., Bahr M., Gößling S., Pischinger S. (2023). Analysis of Ice Formation during Start-Up of PEM Fuel Cells at Subzero Temperatures Using Experimental and Simulative Methods. Energies.

[50] Lan Y., Chen T., Xiao F., Gan Z., Zhang R., Zhang R. (2025). Effects of Initial Water Content of Membrane on Cold Start Performance of PEMFC. Fuel Cells.

[51] Wang J., Shan Z., Wang K. (2025). Sensitivity analysis of the proton exchange membrane fuel cell cold start process with the numerical method. Ionics.

[52] de la Puente M., Gomez A., Laage D. (2025). Why Proton Grotthuss Diffusion Slows down at the Air–Water Interface while Water Diffusion Accelerates. J. Phys. Chem. Lett..

[53] Dasgupta S., Cassone G., Paesani F. (2025). Nuclear Quantum Effects and the Grotthuss Mechanism Dictate the pH of Liquid Water. J. Phys. Chem. Lett..

[54] Mondal A., Ramesh G., Singh R.S. (2022). Manifestations of the structural origin of supercooled water’s anomalies in the heterogeneous relaxation on the potential energy landscape. J. Chem. Phys..

[55] Bergman R., Swenson J. (2000). Dynamics of supercooled water in confined geometry. Nature.

[56] Melillo J.H., Cangialosi D., Di Lisio V., Steinrücken E., Vogel M., Cerveny S. (2024). Complexity of confined water vitrification and its glass transition temperature. Proc. Natl. Acad. Sci. USA.

[57] Davies M.B., Fitzner M., Michaelides A. (2022). Accurate prediction of ice nucleation from room temperature water. Proc. Natl. Acad. Sci. USA.

[58] Senftle T.P., Hong S., Islam M.M., Kylasa S.B., Zheng Y., Shin Y.K., Junkermeier C., Engel-Herbert R., Janik M.J., Aktulga H.M. (2016). The ReaxFF reactive force-field: Development, applications and future directions. npj Comput. Mater..

[59] Kusoglu A., Weber A.Z. (2017). New insights into perfluorinated sulfonic-acid ionomers. Chem. Rev..

